# Gaps and Data Ambiguities in DNA Reference Libraries: A Limiting Factor for Molecular‐Based Biodiversity Assessments Using Annelids as a Case Study

**DOI:** 10.1002/ece3.71544

**Published:** 2025-06-19

**Authors:** Marcos A. L. Teixeira, Eva Aylagas, John K. Pearman, Susana Carvalho

**Affiliations:** ^1^ Biological and Environmental Sciences and Engineering Division (BESE) King Abdullah University of Science and Technology (KAUST) Thuwal Saudi Arabia; ^2^ Cawthron Institute Nelson New Zealand; ^3^ Marine Science Program King Abdullah University of Science and Technology (KAUST) Thuwal Saudi Arabia

**Keywords:** Annelida, Arabian gulf, ARMS, coral reefs, eDNA, Gulf of Oman, metabarcoding, MtCOI‐5P, Red Sea

## Abstract

Regional DNA reference libraries are essential to improve the accuracy of molecular‐based biodiversity assessments, species identification and conservation. However, these libraries are often incomplete, limiting the full potential of molecular tools. In this study, we evaluated the completeness of DNA barcode reference data for annelids from the Red Sea, Arabian Gulf and Gulf of Oman and examined its implications for biodiversity assessments. A database of 2291 worldwide annelid species and 3131–4047 Molecular Operational Taxonomic Units (MOTUs) was compiled from the Barcode of Life Data System (BOLD). Two regional checklists ‐OBIS (498 species) and Wehe & Fiege (892 species)—were cross‐referenced against this database to identify coverage gaps and taxonomic inconsistencies. Only 24% and 23% of species in each checklist, respectively, had corresponding barcodes, and just three species were sampled from the region. Additionally, 43% of BOLD's Barcode Index Numbers (BINs) revealed taxonomic ambiguities. To further assess local annelid biodiversity, we analysed a metabarcoding dataset from 135 Autonomous Reef Monitoring Structures (ARMS) deployed on shallow reefs in the region. This yielded 5375 Amplicon Sequence Variants (ASVs), with 55% classified only to class or phylum level. Of the 1350 MOTUs identified, either none to just 14 species‐level identifications were found depending on the taxonomic classification method and database, 10 of which appear to be cryptic species complexes. Based on proposed MOTU/species ratios, we estimate approximately 992 annelid species in the ARMS dataset, highlighting underexplored regional diversity. Manual inspection of clustered ASVs also revealed potential pseudogene artifacts, taxonomic misidentifications up to the class level and underestimation of species matches due to discordant MOTUs. These findings underscore the urgent need to expand regional reference libraries, apply integrative taxonomy and implement refined, user‐defined MOTU clustering algorithms to improve molecular biodiversity assessments in the region.

## Graphical abstract

Regional DNA reference libraries are crucial for molecular biodiversity assessments, yet they remain incomplete, limiting their effectiveness. Using annelids from the Arabian Peninsula as a case study, we identified considerable gaps in species coverage and minimal representation in public databases. Analysis of metabarcoding data revealed high proportions of low taxonomic levels, misidentifications and possible pseudogene amplifications, underscoring the need for better database curation, updated taxonomic frameworks and improved MOTU clustering methods to enhance biodiversity exploration and conservation efforts.

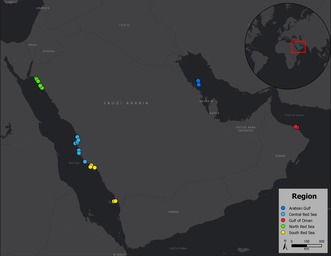

## 1 Introduction

As the ‘rainforests of the sea’ (Reaka‐Kudla 1997), coral reefs host around 25%–33% of marine biodiversity, but are undergoing extensive degradation due to a combination of local and global pressures (Bruno and Selig 2007; Hughes et al. 2017). Coral conservation is fundamental for maintaining regional biodiversity and sustain a variety of ecosystem services, which directly or indirectly benefits one billion people worldwide (Hughes et al. 2007). These ecosystem services include fisheries, shore protection (Sheppard et al. 2005) and tourism, whereas they are a source of pharmaceutical substances produced by reef organisms (Rocha et al. 2011). The resilience and preservation of ecosystem services is affected by alterations in biodiversity (Worm et al. 2006). Thus, it is vital to improve the monitoring and conservation of reef biodiversity, especially of those groups such as benthic invertebrates, that have not been traditionally monitored (Pearman et al. 2016).

The 3D complexity of reef habitats makes the comprehensive assessment of biodiversity challenging and often requires destructive methods. Therefore, research has focused mainly on charismatic and large‐size organisms like corals and fish, that are easy to spot and gain the attention of tourists and managers, whilst the majority of reef biodiversity remains largely unknown (Carvalho et al. 2019; Villalobos, Aylagas, Ellis, et al. 2024, Villalobos, Aylagas, Pearman, et al. 2024). Thus, the knowledge of other groups, such as benthic invertebrates, is often poorer than that of even algae and sponges (e.g., Weigand et al. 2019; Duarte et al. 2020; Leite et al. 2020). Marine benthic invertebrates are characterised by high morphological complexity with studies estimating that most of the diversity is yet to be discovered (Radulovici et al. 2010; Mora et al. 2011). Within marine invertebrates, Annelida are amongst the most phylogenetically diverse groups of organisms, with Polychaeta the most well‐represented class (Ravara et al. 2017; Martin et al. 2021). Annelida are able to thrive in several marine ecosystems including coral reefs (Bailey‐Brock 1999; Hutchings 2008; Carvalho et al. 2019). A recent review (Pamungkas et al. 2019) focused on the discovery progress of Polychaeta based on the WoRMS website, revealing that 11,456 valid species (1417 genera, 85 families) have been named by 835 first authors since 1758. Appeltans et al. (2012) estimated that at least 6320 polychaete species remain to be described and speculated that a total number of species could range between 25,000 and 30,000.

The use of molecular methodologies such as DNA metabarcoding has been increasingly presented as a cost‐efficient approach to describe biological communities and provide diversity metrics comparable to those obtained using traditional methodologies based on the morphological identification of the species (Aylagas et al. 2018; Lanzén et al. 2021; Cote et al. 2023; Basset et al. 2022) with positive impacts in biomonitoring efforts (Leese et al. 2018; Pennisi 2019; Weigand et al. 2019), conservation practices or ecological studies (Delić et al. 2017; Aylagas et al. 2018; Gelis et al. 2021). The high‐throughput identification of multiple species from environmental samples has revolutionised the way we envision monitoring in the near future. The technique can simultaneously identify a wide range of taxonomic groups from the same sample, whilst sample collection is relatively easy and accessible compared to some disruptive methods (e.g., Valentini et al. 2016; Daraghmeh et al. 2025). DNA metabarcoding alone or in combination with DNA barcoding is also helping to unravel hidden cryptic complexes that constitute a substantial fraction of biodiversity (De Luca et al. 2021; Thomasdotter et al. 2023), clarify taxonomic ambiguities (Hebert et al. 2004; Ekrem et al. 2007; Jörger et al. 2012), discover new species (Wehe 2017; Sampieri et al. 2021; Teixeira et al. 2024) or detect potential invasive species (Klymus et al. 2017; Lavrador et al. 2023; Aylagas et al. 2024). However, due to incomplete DNA reference libraries for many tropical coral reefs (Levy et al. 2023; Muenzel et al. 2024), such as those in the Red Sea, and for many groups of organisms, in particular, marine invertebrates (Duarte et al. 2020; Radulovici et al. 2021; Angulo‐Preckler et al. 2024), the applicability of metabarcoding‐based approaches is still limited.

Initial efforts to describe marine benthic communities in the Red Sea using DNA metabarcoding showed a high proportion of non‐assigned molecular taxonomic units at the phylum level (~45%) (e.g., Pearman et al. 2016; Carvalho et al. 2019; Pearman, Chust, et al. 2020a; Pearman, von Ammon, et al. 2020), a pattern commonly found in molecular‐based biodiversity assessments (Canals et al. 2024; Wangensteen et al. 2018). In this context, updated and curated regional DNA barcoding reference libraries are fundamental to maximise the potential of DNA metabarcoding to address biodiversity‐related questions. Whilst building DNA reference libraries and their continuous improvement is critical, a thorough revision of the current status of local DNA libraries is necessary to optimise efforts (Weigand et al. 2019; Radulovici et al. 2021).

Here, we aim to (1) perform a barcode availability and a quality assessment of the current publicly available mitochondrial cytochrome oxidase I (COI) sequences for Annelida within the Barcode of Life Data System (BOLD; Ratnasingham and Hebert 2007); (2) perform a gap analysis for Annelida species reported in the Red Sea, Arabian Gulf and Gulf of Oman, including reports from the OBIS database and the extensive checklist compiled by Wehe and Fiege (2002); (3) based on a metabarcoding dataset obtained from a total of 135 Autonomous Reef Monitoring Structures (ARMS) deployed at 28 coral reefs mainly along the Red Sea, but also in the Arabian Gulf and Gulf of Oman, obtain amplicon sequence variants (ASVs) and estimate Molecular Operational Taxonomic Units (MOTUs). An assessment of the total number of species using ‘MOTU/species’ ratios obtained from the dataset generated in point 1 will be made, and species‐level identifications (including their current taxonomic status) by using RDP, MEGAN, and a manual ID blast comparison against a regional dataset (Indian Ocean; MetaZooGene Atlas & Database) will be attempted; and (4) to explore taxonomic ambiguities in the obtained molecular‐based assignments and current metabarcoding limitations based on the previous points. This approach highlights the existing knowledge gaps, bioinformatic limitations and the many taxonomic revisions still pending in the region.

## 2 Material and Methods

### 2.1 Data Mining and BOLD Dataset Creation

**FIGURE 1:**
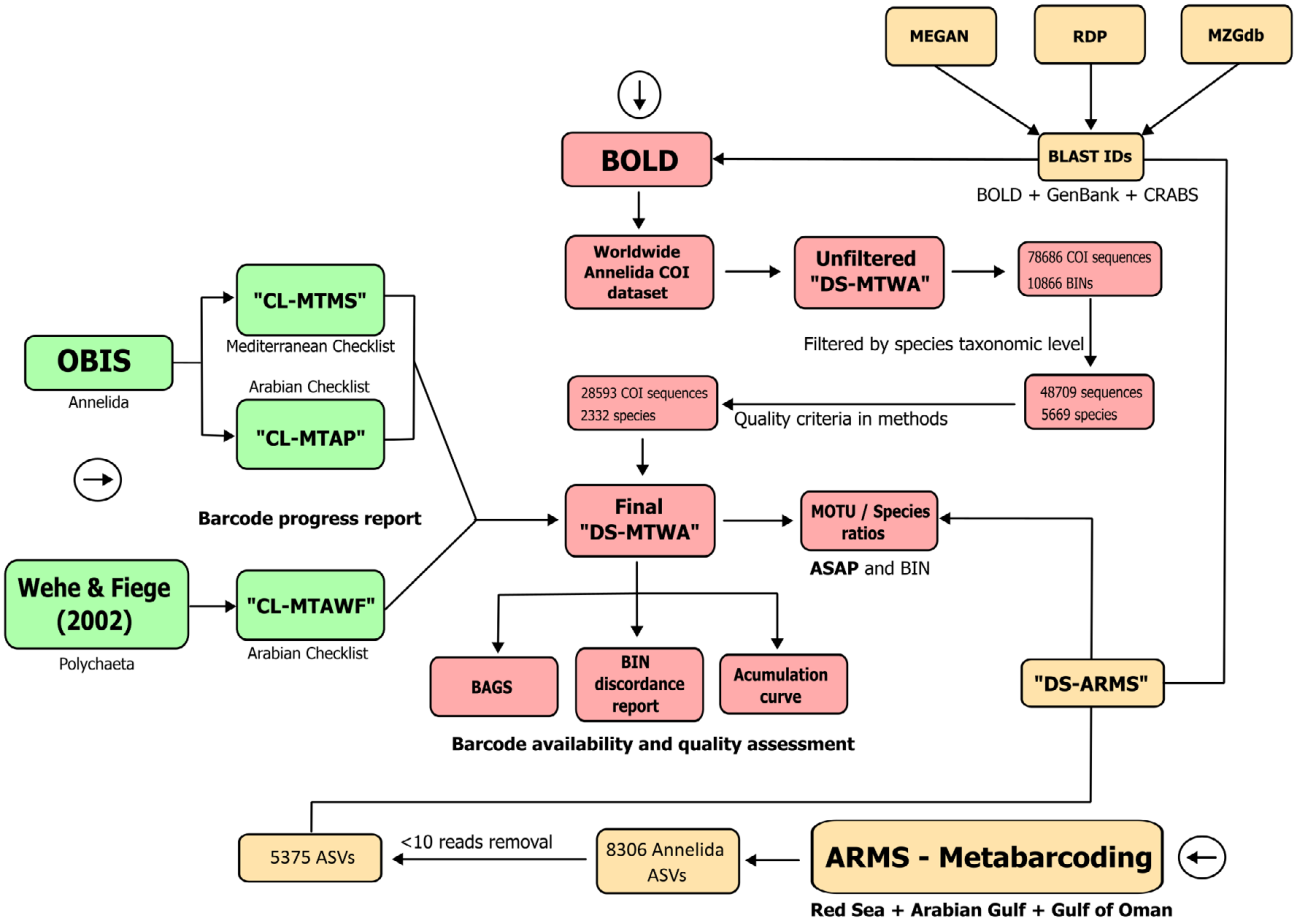
Schematic representation for the methodology described in this study.

BOLD platform was used to search for all publicly available mtCOI‐5P sequences belonging to Annelida, which also includes records automatically mined from GenBank. Using this data, the dataset DS‐MTWA was created (DOI: https://doi.org/10.6084/m9.figshare.27938121; on 10‐July‐2023) for downstream analysis. A species was considered successfully barcoded if at least one mtCOI‐5P sequence (> 300 bp) was available. MtCOI‐5P sequences without information on species name, lacking Barcode Index Numbers (BINs), flagged for contamination, stop codons or indels and with tag codes in the taxonomic identification were disregarded. Tag codes (e.g., ‘*Tharyx* sp. CIRR‐IK‐2019‐1’) are often used either to distinguish different lineages within cryptic complexes or between different populations in certain BOLD projects to make use of the several available analytical tools in the platform. However, this can overestimate the number of available species and the number of discordant BIN records, the latter explained in the next methodology section. The initial dataset contained 78,686 published records forming 10,866 BINs (clusters), with specimens from 161 countries. Out of these records, only 48,709 (62%) had species names and represent 5669 different species. Filtering the database based on the criteria mentioned above, a final dataset (DS‐MTWA) was obtained, that included 29,593 DNA barcodes (18,463 mined from GenBank) from 2332 species. This dataset was further clustered into MOTUS by using an additional alternative method to BINs (see Section 2.5). Using this dataset as a baseline, the entire methodology is summarised in Figure 1 and described below in more detail.

### 2.2 Regional Checklists

Two lists of Annelida records were downloaded from the Ocean Biodiversity Information System (OBIS; https://mapper.obis.org/) on 21‐June‐2023 for: (1) Red Sea, Arabian Gulf and Gulf of Oman (these Ocean regions henceforth referred to ‘Arabian Peninsula’ for simplicity), which is the main region of interest for this study; (2) the Mediterranean Sea, for comparison purposes and proximity. The records were filtered to the species level only and, subsequently, taxa with an unclear taxonomic status (*nomen nudum, interim* unpublished, temporary name, uncertain, *taxon inquirendum*) were excluded. Alternative representations of names were treated as objective synonyms. Filtered lists were kindly provided by Dr. Sarah Faulwetter (University of Patras, Greece). Taxonomy used followed the World Polychaeta Database (WoRMS; https://www.marinespecies.org/; Read and Fauchald 2020) and, as such, some taxa are not assigned to any class (treated here as incertae sedis). The former Annelida Class Sipuncula (now Order Sipuncula) was treated as a major group for comparison purposes. The Arabian checklist (CL‐MTAP; publicly available at DOI: https://doi.org/10.6084/m9.figshare.27938121) comprises 2 classes, 10 orders, 58 families, 235 genera and 498 species, whereas the Mediterranean one (CL‐MTMS; publicly available at DOI: https://doi.org/10.6084/m9.figshare.27938121) comprises 2 classes, 13 orders, 74 families, 444 genera and 1114 species. Both species lists were uploaded to BOLD (Ratnasingham and Hebert 2007; https://www.boldsystems.org) and are publicly available for all BOLD users. Furthermore, the detailed Arabian checklist compiled by Wehe and Fiege (2002) was also digitised and similarly analysed in BOLD. This list is focused on Polychaeta, comprising 62 families and 334 genera, 788 species, 16 subspecies and 3 species complexes. After updating the list based on WoRMS, the final checklist comprised 7 orders, 59 families and 380 genera, 884 species and 8 subspecies (CL‐MTAWF; publicly available at DOI: https://doi.org/10.6084/m9.figshare.27938121 and for all BOLD registered users).

### 2.3 BOLD Barcode Completion, Availability and Quality

A global gap analysis was conducted by comparing the publicly available barcoded species of Annelida (DS‐MTWA; 2332 species), as of 10 July 2023, with the total number of valid species reported in the literature (e.g., Weigand et al. 2019; Leite et al. 2020; Duarte et al. 2020). Regional checklists from the Arabian Peninsula (CL‐MTAP) and the Mediterranean Sea (CL‐MTMS) were compared against the mtCOI‐5P sequence records available in the DS‐MTWA dataset using the BOLD checklist tool, in order to obtain the percentage of barcoded taxa. Also, the BOLD accumulation curve tool was used to visualise trends in the total number of sequences, species and BINs over time, for the whole phylum and for each family.

All species included in the dataset (DS‐MTWA; i.e., worldwide Annelida BOLD dataset) were associated with a BIN (Ratnasingham and Hebert 2013). These BINs were annotated (as of 20 July 2023) using the BOLD's BIN discordance report tool and categorised into one of three possible taxonomic congruence grades: Discordant (i.e., more than one nominal species assigned to the same BIN), Concordant (i.e., one species assigned to a single BIN; same species may have different BINs) and Singletons (nominal species with just one available sequence). The BIN system, unique to BOLD, clusters COI sequence data into MOTUs independent of prior taxonomic assignment, using the Refined Single Linkage (RESL) algorithm. As such, it allows confirmation between barcode sequence clusters versus species designations concordance. This validation is performed by comparing the taxonomy of the input records against all other records within the same BINs, including those submitted and curated by other users (Ratnasingham and Hebert 2007). The dataset DS‐MTWA was also analysed using the Barcode, Audit & Grade System (BAGS) rating tool (Fontes et al. 2021), assessed via its web interface (https://github.com/tadeu95/BAGS), as of 24 July 2023. For large datasets such as the ones used in our study, BAGS must be run in the R Software (2023.06.1, Build 524) following the authors' guidelines provided in the ‘BAGS_run.R’ section of the website. The assignment of each grade is based on the quality, availability and replicability of the data and metadata for each species, as well as the quality and congruence of the mtCOI‐5P sequences, evaluated in accordance with their BINs. The grades are attributed to each species according to the following criteria: Grade A (consolidated concordance), where the morphospecies is assigned a unique BIN (also uniquely assigned to that species), and the species has more than 10 specimens present in the library; Grade B (basal concordance), similar to Grade A, but the species has 10 or fewer specimens present in the reference library; Grade C (multiple BINs), where the morphospecies is assigned to more than one different BIN, but each of those BINs is exclusively assigned to that species; Grade D (insufficient data), where the species is not assigned discordantly, but it has less than three specimens available in the reference library; Grade E (discordant species), where a species is assigned to a BIN that also contains sequences from more than one different species. This may indicate taxonomic inconsistencies, potential misidentifications or evidence of paraphyly or polyphyly.

### 2.4 ARMS Metabarcoding Dataset and ASVs Taxonomic Assignments

**FIGURE 2:**
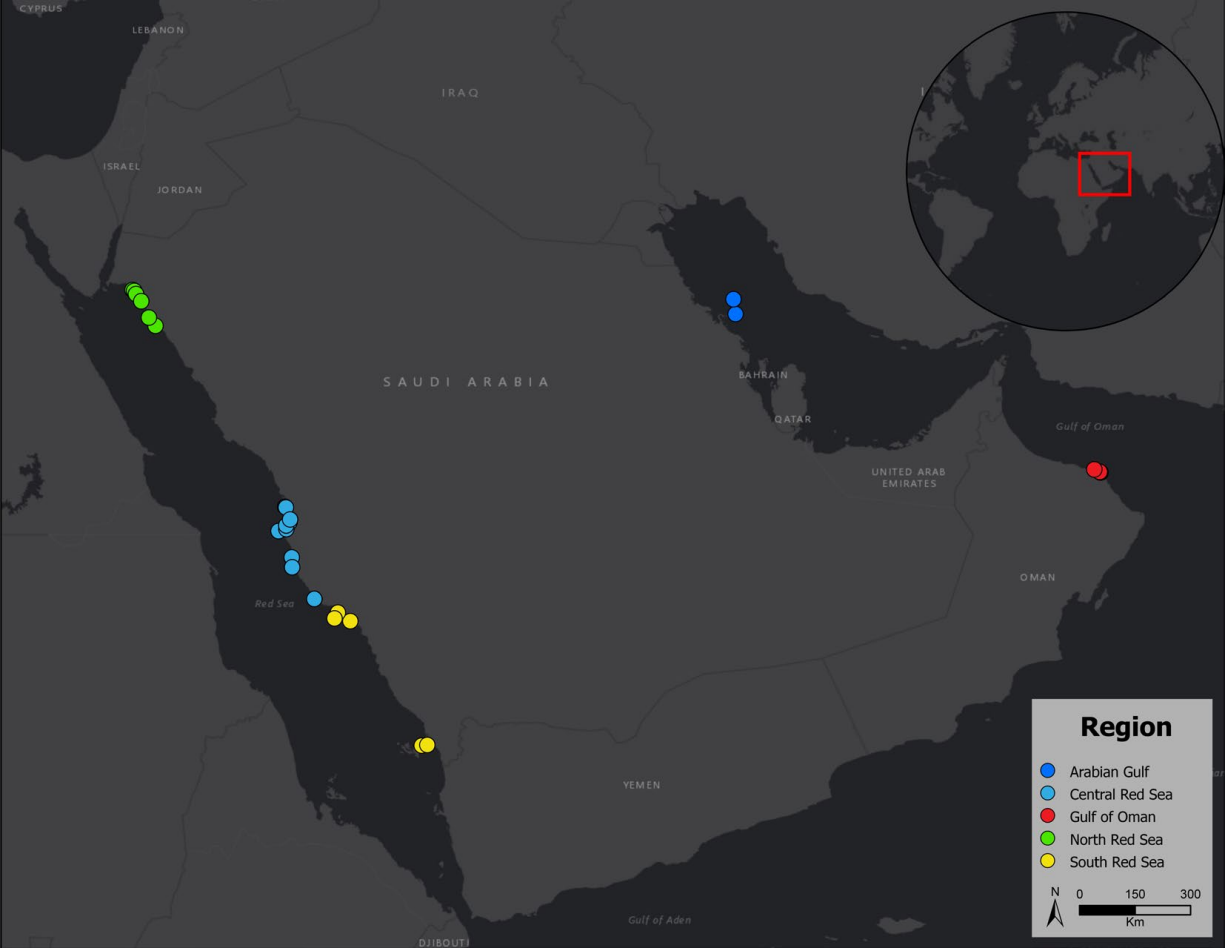
Map of the Arabian Peninsula showing the different locations of ARMS retrieval. Each colour corresponds to a major sampling region (North, Central and South Red Sea, Oman and Arabian Gulf).

Triplicate Autonomous Reef Monitoring Structures (ARMS) consisting of nine 22.5 × 22.5 cm stacked PVC plates were deployed on 28 reefs between 2014 and 2019, along the North, Central and Southern Saudi Red Sea coast, the Arabian Gulf and the Gulf of Oman (Table 1; Figure 2). Survey sites ranged either from Inshore or from ~300 m to ~70 km from shore and were classified into three categories according to their position in the continental shelf following Khalil et al. (2017): (i) nearshore reefs (nearest to shore and with adjacent waters approximately 20 m deep); (ii) midshore reefs (located between nearshore and offshore reefs, characterised by neighbouring waters ranging from 50 to 200 m deep) and (iii) offshore reefs (farther apart from shore and surrounded by waters deeper than 200 m). Environmental conditions for each reef are detailed in Table 1. At a subset of reefs in the Red Sea, ARMS were replaced and redeployed, totalling 135 units. Each unit was deployed at 8–10 m depth on the hard framework of the reefs for a period between 1 and 3 years. Each ARMS was retrieved by placing a box on top of the ARMS structure to avoid loss of mobile communities and was placed in a large container with local filtered seawater for transportation.

**TABLE 1:** Number and year of ARMS retrieval, with the respective coordinates, reefs and regions.

Reef code	Region	Country	Coordinates		Depl. date	#ARMS	D	CC	Shelf position	Exposure
			Lat	Long						
DR06	NRS	Saudi Arabia	27.073328	35.787456	2016	3	10	22.05%	Offshore	Semi
DR07	NRS	Saudi Arabia	27.274417	35.626496	2016	3	10	20.17%	Midshore	Protected
DR07	NRS	Saudi Arabia	27.274417	35.626496	2018	3	10	16.60%	Midshore	Protected
DR08	NRS	Saudi Arabia	27.943078	35.232253	2016	3	10	25.50%	Inshore	Protected
DR09	NRS	Saudi Arabia	27.921	35.269	2016	3	10	32.16%	Inshore	Protected
DR10	NRS	Saudi Arabia	27.844433	35.312891	2016	3	10	28.32%	Midshore	Protected
DR12	NRS	Saudi Arabia	27.670733	35.440029	2016	3	10	12.11%	Midshore	Protected
DR12	NRS	Saudi Arabia	27.670733	35.440029	2018	3	10	11.54%	Midshore	Protected
AMF	CRS	Saudi Arabia	22.089319	38.778075	2015	3	10	NA	Offshore	Exposed
AMF	CRS	Saudi Arabia	22.089319	38.778075	2017	3	10	30.3%	Offshore	Exposed
AMF	CRS	Saudi Arabia	22.089319	38.778075	2019	3	10	32.29%	Offshore	Exposed
ASA	CRS	Saudi Arabia	22.299799	39.044185	2015	3	10	NA	Midshore	Semi
ASA	CRS	Saudi Arabia	22.299799	39.044185	2017	3	10	13.36%	Midshore	Semi
ASHO	CRS	Saudi Arabia	22.299799	39.044185	2019	3	10	10.09%	Midshore	Semi
ASF	CRS	Saudi Arabia	22.138715	38.967578	2015	3	10	NA	Midshore	Semi
ASF	CRS	Saudi Arabia	22.138715	38.967578	2017	3	10	17.9%	Midshore	Semi
AFL	CRS	Saudi Arabia	22.223781	38.964983	2015	3	10	NA	Midshore	Exposed
AFL	CRS	Saudi Arabia	22.223781	38.964983	2017	3	10	20.42%	Midshore	Exposed
AFHL	CRS	Saudi Arabia	22.223781	38.964983	2019	3	10	22.35%	Midshore	Exposed
AWN	CRS	Saudi Arabia	22.679196	38.940043	2016	3	10	17.06%	Midshore	Semi
AWS	CRS	Saudi Arabia	22.661607	38.958546	2015	3	NA	NA	NA	NA
JD01	CRS	Saudi Arabia	21.452855	39.111838	2014	3	10	20.17%	Midshore	Semi
JD01	CRS	Saudi Arabia	21.452855	39.111838	2017	3	10	17.22%	Midshore	Semi
JD02	CRS	Saudi Arabia	21.082017	39.20105	2014	3	10	6.45%	Nearshore	Semi
JD02	CRS	Saudi Arabia	21.082017	39.20105	2017	3	10	13.97%	Nearshore	Semi
JD02	CRS	Saudi Arabia	21.082017	39.20105	2019	3	10	9.33%	Nearshore	Semi
JD03	CRS	Saudi Arabia	21.22495	39.120773	2014	3	10	6.38%	Nearshore	Semi
JD03	CRS	Saudi Arabia	21.22495	39.120773	2017	3	10	13.97%	Nearshore	Semi
JD03	CRS	Saudi Arabia	21.22495	39.120773	2019	3	10	21.42%	Nearshore	Semi
KAEC	CRS	Saudi Arabia	22.369847	39.060165	2015	3	10	NA	Nearshore	Semi
AN	CRS	Saudi Arabia	20.4961672	39.6354076	2017	3	10	NA	Nearshore	Exposed
ALR5	CRS	Saudi Arabia	20.4961672	39.6354076	2019	3	10	13.59%	Nearshore	Exposed
ALR3	CRS	Saudi Arabia	19.90149	40.52464	2017	3	10	0.47%	Nearshore	Protected
SA	SRS	Saudi Arabia	19.90149	40.52464	2019	3	10	0.65%	Nearshore	Protected
CG	SRS	Saudi Arabia	20.11	40.22	2017	3	10	0%	Nearshore	Protected
F18	SRS	Saudi Arabia	16.88	42.26	2017	3	10	NA	Nearshore	Semi
F24	SRS	Saudi Arabia	16.79	42.199	2017	3	NA	NA	NA	NA
FS11	SRS	Saudi Arabia	16.89465	42.39625	2019	3	10	0.17%	Nearshore	Protected
WS	SRS	Saudi Arabia	20.121593	40.218074	2017	3	10	1.63%	Nearshore	Protected
ALR7	SRS	Saudi Arabia	20.121593	40.218074	2019	3	10	2.81%	Nearshore	Protected
JJL	Arabian Gulf	Saudi Arabia	27.3599667	49.8885	2019	3	NA	NA	NA	NA
JKI	Arabian Gulf	Saudi Arabia	27.7193667	49.8379667	2019	3	NA	NA	NA	NA
BK1	Gulf of Oman	Oman	23.5120556	58.7601944	2019	3	NA	NA	NA	NA
BK2	Gulf of Oman	Oman	23.5274167	58.739889	2019	3	NA	NA	NA	NA
CAT1	Gulf of Oman	Oman	23.5859167	58.6097778	2019	3	NA	NA	NA	NA

*Note:* Environmental data also added when available.

Abbreviations: CC, coral cover; CRS, Central Red Sea; D, depth; NRS, North Red Sea; SRS, South Red Sea.

In the laboratory, each ARMS was disassembled and samples processed as described in Leray and Knowlton (2015). In brief, after photographing each plate surface, sessile organisms were scrapped from each plate, blended and stored in 96% ethanol for further analysis. Water from each container was poured over a stack of three sieves to collect three mobile size fractions (106, 500 and 2000 μm). Each biological fraction was processed for DNA metabarcoding analysis (Leray and Knowlton 2015). Genomic DNA was extracted and eukaryotic sequences targeted using primers designed to amplify a 313 bp fragment of the COI gene with a versatile primer set (Leray et al. 2013) using the PCR conditions detailed in Leray and Knowlton (2015). PCR amplicons were prepared for Illumina MiSeq sequencing, undertaken at the King Abdullah University of Science and Technology (KAUST) Bioscience Core Laboratory. The reads generated on the Illumina MiSeq were processed following Pearman, Chust, et al. (2020). Briefly, reads were demultiplexed on the MiSeq platform, and primer sequences were trimmed using *cutadapt* (Martin 2011). Reads were truncated at 165 bp (forward) and 160 bp (reverse) and filtered based on maximum expected errors (maxEE = 4 for forward, 6 for reverse) using the DADA2 pipeline (Callahan et al. 2016) in R (R Core Team 2018). Reads were dereplicated, singletons discarded and ASVs inferred. Paired‐end reads were merged with a minimum 10 bp overlap and chimeras were removed using *removeBimeraDenovo*. ASV sequences were translated and aligned to the MIDORI database (Machida et al. 2017) using MACSE (Ranwez et al. 2011), with sequences containing stop codons considered as pseudogenes and removed from further analysis. Taxonomic classification was performed using the RDP naïve Bayesian classifier method described in Wang et al. (2007) against a curated BOLD database (Ratnasingham and Hebert 2007) supplemented with COI sequences from NCBI (https://www.ncbi.nlm.nih.gov/) and CRABS (Jeunen et al. 2022), using a confidence threshold of 0.51. Only ASVs classified as eukaryotes were retained for further analysis.

The detailed number of reads per ASV is provided in Table S1. Raw sequencing data were deposited in the SRA repository under the Accession Numbers: PRJNA485689, PRJNA1063439 and at Figshare with the following doi's: https://doi.org/10.6084/m9.figshare.5549365; https://doi.org/10.6084/m9.figshare.5552782; https://doi.org/10.6084/m9.figshare.5552932. After filtering for Annelida only, an initial dataset of 8306 ASVs was obtained. Upon removing ASVs with < 10 reads, the final ARMS dataset contained a total of 5375 ASVs, referred to from now on as ‘DS‐ARMS’ (Figure 1; ASV alignment available at: https://doi.org/10.6084/m9.figshare.27938121). ASVs from the DS‐ARMS dataset were further clustered into MOTUS (see below section).

### 2.5 MOTU Clustering and MOTU/Species Ratios

In addition to BINs, which are unique to sequences deposited in BOLD and therefore not directly comparable with metabarcoding‐generated data (i.e., ASVs), an alternative delineation method was applied to cluster ASVs (DS‐ARMS) and barcodes from the BOLD dataset (DS‐MTWA) into MOTUs (Figure 1). The Assemble Species by Automatic Partitioning (ASAP, Puillandre et al. 2021) algorithm was selected for its efficiency, ease of use and its more conservative approach to species delimitation compared to BINs. This analysis was performed via the command line on a Unix‐based operating system for the BOLD Annelida COI dataset (DS‐MTWA; large dataset, total of 29,547 barcodes) and in a web interface (https://bioinfo.mnhn.fr/abi/public/asap/asapweb.html) for the ASVs dataset (DS‐ARMS; short dataset, total of 5375 ASVs) with default settings using the Kimura (K80) distance matrix. The obtained top 10 results were selected, with the chosen partition based on the lowest score as recommended by Puillandre et al. (2021). It is well known that MOTUs do not always correspond directly to morphological species (e.g., Teixeira, Vieira, et al. 2022; Vieira et al. 2019). In some cases, multiple MOTUs represent a single species (e.g., *Hediste diversicolor* (O.F. Müller, 1776), Teixeira, Bakken, et al. 2022), whilst in others, a single MOTU comprises two or more formal species (e.g., *Platynereis agilis* (Keferstein, 1862) and *P. massiliensis* (Moquin‐Tandon, 1869), Teixeira, Langeneck, et al. 2022). To minimise this inconsistency and estimate an accurate representation of species when only molecular data is available, MOTUs/species ratios were calculated for the ‘DS‐MTWA’ dataset. This dataset consists exclusively of sequences identified to the species level, providing a robust reference pool for estimating ratios between observed MOTUs and known species. Accordingly, the MOTU/species ratios derived from the DS‐MTWA dataset, using both BIN and ASAP delineation methods, were then used to estimate the number of species represented in the metabarcoding dataset DS‐ARMS using the formula: *X* = (No. ASAP MOTUs ‘DS‐ARMS’ × No. Species ‘DS‐MTWA’)/No. ASAP MOTUs ‘DS‐MTWA’. This approach allows for an informed approximation of species richness within the metabarcoding dataset based on public reference data.

### 2.6 Species‐Level Identifications

In addition to the RDP classifier mentioned above, species‐level taxonomic assignments for ASVs in the DS‐ARMS dataset were further inferred using BLAST (parameters: *‐max_target_seqs 10 ‐qcov_hsp_perc 90 ‐perc_identity 95*). The queries were matched against a custom COI BLAST database, as previously detailed in the ARMS metabarcoding dataset section. BLAST results were subsequently parsed in MEGAN (Huson et al. 2007), applying a minimum similarity threshold of 95% and a minimum query cover of 90% for taxonomic assignment. In addition, the MetaZooGene Atlas and Database (MZGdb; https://metazoogene.org/mzgdb/; O'Brien et al. 2024) was also used to search for potential species‐level identifications in the DS‐ARMS dataset. COI sequences of Annelida restricted to species‐level records from the Indian Ocean were downloaded for this purpose. This database is an open‐access portal for data and metadata, synchronised with the NCBI GenBank and BOLD data repositories. The initial MZGdb alignment contained 6972 sequences, which was reduced to 6594 after filtering out records that did not correspond to the barcode region, were too short (< 100 bp) to align with our ASVs and/or were too divergent for reliable phylogenetic reconstruction and manual blast comparison. The sequences within this dataset were further trimmed to match ASV length and, together with the DS‐ARMS dataset, were aligned using the MAFFT online server (ver. 7.0, see https://mafft.cbrc.jp/alignment/server/; Katoh and Standley 2013) and the resulting ASV alignments (with and without inclusion of the MZGdb dataset) are available on Figshare (DOI: https://doi.org/10.6084/m9.figshare.27938121). A Neighbour‐Joining (NJ) phylogenetic tree using the Kimura 2‐parameters model was built using MEGA software (Molecular Evolutionary Genetic Analysis, ver. 7, see https://www.megasoftware.net/; Kumar et al. 2016). This tree was used to visually summarise the manual comparison between DS‐ARMS and MZGdb sequences. Clades with < 5% K2P divergence were considered to represent the same species, and species‐level identification, where available, was summarised accordingly.

All obtained species‐level identifications were aligned with our ASVs and with sequences of the same species from the previously mentioned custom databases (‘ASV‐complex’: available in figshare: https://doi.org/10.6084/m9.figshare.27938121). These alignments were used to construct a NJ phylogenetic tree based on the Kimura 2‐parameter model, using MEGA software to investigate potential species complexes. For each species‐level assignment, a tentative ecological status [categorised as cryptogenic, native, unassigned (e.g., undescribed species complexes) based on Carlton and Schwindt 2024] was proposed based on the type locality, the region of the blast match from public databases and the known geographic distribution of the assigned ASV. The corresponding GenBank accession numbers, BOLD and CRABS IDs are detailed in Table S2, along with the respective references.

## 3 Results

### 3.1 Analysis of the Annelida Dataset From BOLD

#### 3.1.1 Barcode Availability

**FIGURE 3:**
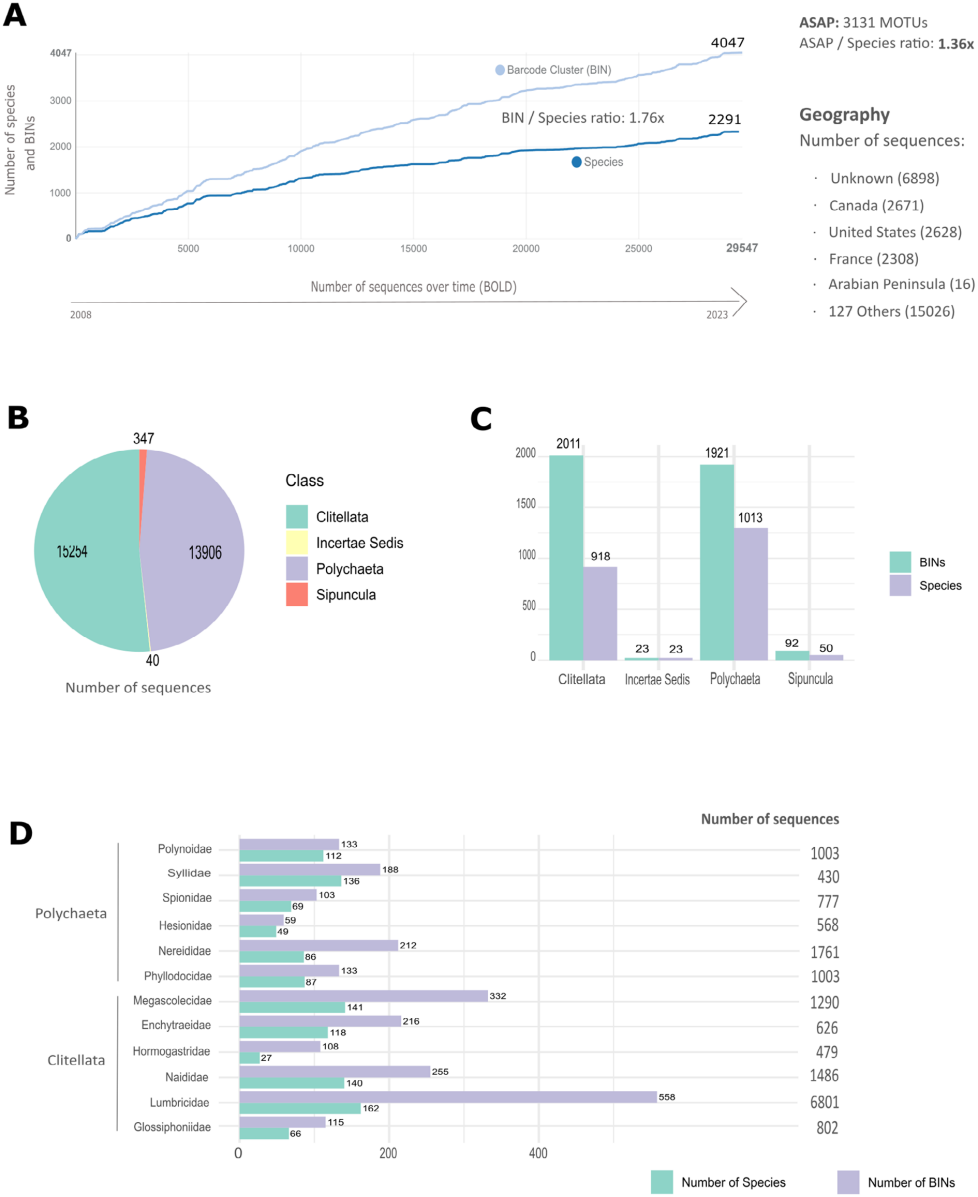
(A) Accumulation curve using all records from the dataset DS‐MTWA (Figure 1). The number of species and number of BINs by number of public sequences submitted to BOLD over time from 2008 to 2023. Total number of sequences for the most well represented regions (‘Others’, corresponding to other locations not individually mentioned), as well as for the Arabian Peninsula and number of MOTUs based on ASAP and respective species ratio are also displayed. (B) The number of available sequences per class (Polychaeta and Clitellata), within the Sipuncula (order, former Annelida class) and assigned to no class (incertae sedis). (C) Number of species and BINs (MOTUs) within each Class, the Sipuncula and incertae sedis. (D) The number of species, BINs, total number of sequences for the most represented families in the dataset.

A total of 2291 species of Annelida were retrieved from BOLD, comprising 29,547 public sequences in the DS‐MTWA dataset, which resulted in 4047 BINs (Figure 3A). The dataset also generated between 2768 and 3131 ASAP clusters, with the optimal partition corresponding to 3131 monophyletic clades exhibiting low genetic divergence (< 3% K2P). Most sequences belonged to the Class Clitellata (51.5%; primarily terrestrial and freshwater), followed by Polychaeta (47%; predominantly marine). The former Class Sipuncula accounted for 1.2% of sequences, whereas 0.1% of the records are assigned to incertae sedis, indicating unresolved classification at the class level (Figure 3B). Although the Class Polychaeta had a higher number of represented species (1300; 57%) and families (73; 61%) than Clitellata (922 species, 40%; 36 families, 30%), the latter presented a slightly higher proportion of BINs (88%) compared to the former (84%) (Figure 3C).

Family Lumbricidae (Clitellata) contributed the most (23%) to the total number of sequences, followed by Nereididae (Polychaeta, 6%), Naididae (Clitellata, 5%), Megascolecidae (Clitellata, 4.4%), Phyllodocidae and Polynoidae (Polychaeta, 3.4% each). All remaining families displayed in Figure 3D represented 12.3%, whereas the 107 families not displayed contributed to 42.5% of sequences. Despite having the lowest number of available sequences amongst the most well represented families (430, 1.5%), Syllidae (Polychaeta) held a similar number of barcoded species (136, 5.9%) as the Megascolecidae (141, 6.2%). The latter is second only to Lumbricidae (162 barcoded species, 7.1%) (Figure 3D).

As for the biogeographic distribution, 6898 records in the dataset lack georeferenced coordinates (Figure 3A), with most sequence records coming from Europe (5514), followed by North America (5304) and Southeast Asia (1891). Only 16 sequences were retrieved from the Arabian Peninsula, corresponding to three species *Hydroides elegans* (Haswell, 1883), *Hydroides operculata* (Treadwell, 1929) and *Archinome jasoni* (Borda et al. 2013), mostly from the Arabian Sea. No DNA barcodes with species‐level identifications were recorded from the Red Sea.

#### 3.1.2 Barcode Quality Assessment

From a total of 4047 BINs (Figure 3A, Table 2), 51% showed no apparent taxonomic conflict (i.e., concordant) and 6% presented discordance. A large portion of the BINs (43%) were singletons, having only one available sequence. Similar patterns were found for Polychaeta and Clitellata, with more concordant (8 to 9 times) than discordant BINs. This analysis also revealed that the global number of BINs was almost twice the number of species (1.76 times, Figure 3A). The total number of MOTUs (ASAP) was more conservative (3131; 1.36 times the number of species). In general, within the two most well represented groups, the Clitellata seems to be the most affected by excess of BINs in relation to the number of species (2.2 times), whereas Polychaeta showed a ratio of 1.5 (Figure 3C). Looking further into the most well represented families in the dataset, Lumbricidae (Clitellata) has the highest number of BINs and barcoded species, whilst Hesionidae (Polychaeta) is the least represented, with a similar amount of species and BINs amongst the highlighted families (Figure 3D). Within Polychaeta, the Nereididae appear to be the most notable example of excess BINs compared to the number of species (around 2.5 ratio) (Figure 3D).

**TABLE 2:** BIN discordance report from BOLD analytics and BAGS rating results (see Section 2) for all the species using the dataset DS‐MTWA, with both a global view (Annelida) and within each major taxonomic group.

	Concordant	Discordant	Singleton	BAGS species rating (%)				
				A	B	C	D	E
Polychaeta	1023	116	782	7.9	16.5	15.4	35.2	25.
Clitellata	1004	111	896	3.5	10.9	20.1	38.9	26.6
Sipuncula	29	6	57	0	4.0	22.0	44.0	30.
incertae sedis	7	6	10	4.4	13.0	0	73.9	8.7
Annelida	2063	239	1745	5.9	13.9	17.3	37.2	25.7

Following the BAGS approach (Table 2), the Annelida dataset revealed very low percentages of concordant species with more than three available sequences (Grades A + B, 19.8%). Only 453 of the species were assigned to a unique BIN, with this BIN also assigned uniquely to that species. BAGS also showed the percentage of potential species complexes (represented by Grade C), where the same morphospecies is assigned to multiple BINs. In total, 397 species might belong to undescribed cryptic lineages requiring further taxonomic examination and formal description. Grade C values are the highest amongst the Clitellata (up to 20%). A large portion of the Annelida dataset (63%) either has species with less than three available sequences (Grade D, 853 species) or with a discordant species assignment (Grade E, 588 species), the latter with ambiguities found in the taxonomic identifications for these records. Most of the available data on the incertae sedis group belongs to Grade D, with 17 out of 23 species having less than three available sequences.

#### 3.1.3 Barcode Progress Report

**FIGURE 4:**
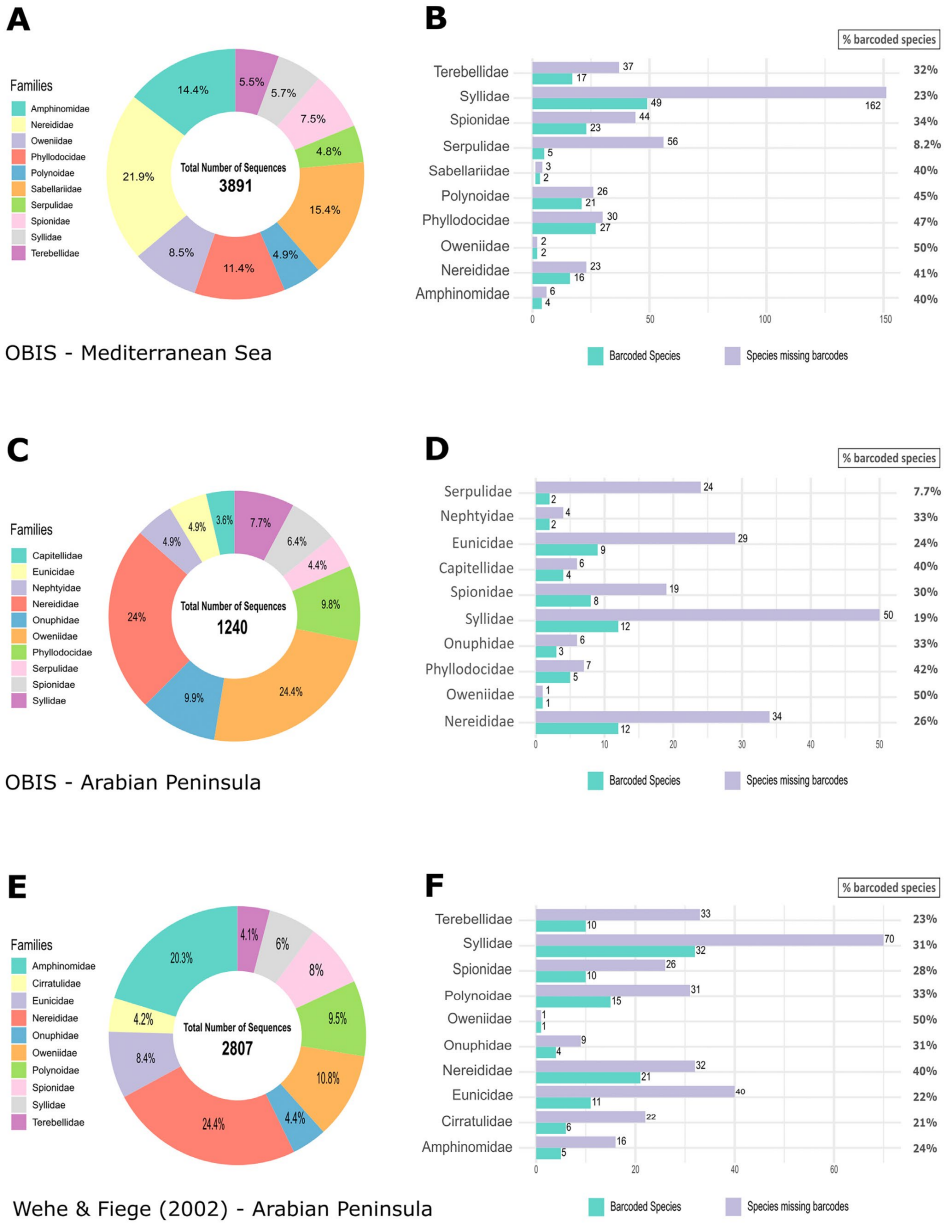
The number of sequences and barcoded species for the 10 most represented Families. (A, B), using the OBIS Annelida checklist for species reported in the Mediterranean Sea (CL‐MTMS). (C, D) using the OBIS Annelida checklist for species reported in the ‘Arabian Peninsula’ (CL‐MTAP). (E, F) using the Polychaeta checklist by Wehe and Fiege (2002), digitised on BOLD and updated using WoRMS (CL‐MTAWF) for species reported in the Arabian Peninsula.

The number of DNA barcodes assigned to different taxa levels amongst the Annelida is highly variable (Table 3). Only 24% of the species (121), 40.85% of the genera (96) and 67.24% of the families (39) from the Arabian Peninsula checklist (CL‐MTAP) had DNA barcodes. Within the most represented families (Figure 4C), the Family Nereididae, despite having the second highest number of sequences (298, 24%) showed one of the lowest levels of completion (26%; Figure 4D), with Serpulidae and Syllidae showing the lowest (7.7%) and second lowest (19%) levels of completion, respectively (Figure 4D). As expected, the families with a smaller number of reported species for the Arabian region are also the ones showing the highest levels of completion in the database, with up to 50% of the species having DNA barcodes (e.g., Oweniidae, with just two reported species). The lack of knowledge may be better assessed if considering the extremely disparate number of valid taxa of these families reported for the Arabian region in OBIS: 62 for Syllidae, 46 for Nereididae, 26 for Serpulidae, but only six for the Nephtyidae and two for the Oweniidae.

**TABLE 3:** The number and percentages of barcoded species, genera and families from the Annelida Mediterranean checklist (CL‐MTMS; OBIS), Arabian checklist (CL‐MTAP, OBIS) and the Arabian checklist from Wehe and Fiege (2002; updated based on WoRMS: CL‐MTWAF) based on available DNA barcodes in the BOLD database.

Regional checklists	No. species	No. genus	No. families	% barcoded (BOLD dataset DS‐MTWA)		
				Species	Genus	Family
OBIS (Mediterranean)	1114	444	74	30	45	73
OBIS (Arabian Peninsula)	498	235	58	24	41	67
Wehe and Fiege (2002); Arabian Peninsula	807	334	62	—	—	—
Wehe and Fiege (2002); Arabian Peninsula, CL‐MTWAF	892	380	59	23	37	75

Focusing on Polychaeta and using the Wehe and Fiege (2002) checklist as a reference, a similar percentage of barcoded species (23%) was observed compared to the OBIS dataset, though this corresponds to a higher absolute number of species (203). In addition, 37% of the genera (140) and 75% of the families (44) listed in the checklist have associated DNA barcodes (Table 3). Amongst the best represented families, excluding Oweniidae, which only included two reported species but has a high number of available sequences, the Nereididae (40%), Polynoidae (33%) and Syllidae (31%) exhibit the highest proportions of barcoded species (Figure 4F). Despite Amphinomidae having the second highest number of publicly available sequences (570), only five of the 21 species reported in the study region are barcoded (Figure 4F). However, this family is much better represented in the Wehe and Fiege (2002) checklist when compared to the OBIS dataset.

For comparison purposes, the same gap analysis was done for the Mediterranean Sea using OBIS (CL‐MTMS), with the total number of reported taxa for this region being much higher, with more than double the Annelida species (1114) recorded for the Arabian Peninsula (498). For the Mediterranean, only 30% of the species (300), 45% of the genera (200) and 73% of the families (54) were represented by barcodes (Table 3). Amongst the 10 most represented families, the Nereididae have the highest number of available sequences (854, 15.7%), with Sabellariidae (599, 11%) and Amphinomidae (561, 10.3%) also being well represented (Figure 4A,B). On the other end, Serpulidae and Syllidae were again at the lowest end regarding barcoded species, with 8.2% and 23%, respectively (Figure 4B). However, these families are within the top three of the most diverse ones reported for the Mediterranean Sea (211 species for Syllidae, 67 for Spionidae and 61 for Serpulidae; Figure 4B).

### 3.2 ARMS Metabarcoding Dataset Analysis

The dataset ‘DS‐ARMS’ contained 5375 ASVs, comprising eight orders (seven within Class Polychaeta and one Clitellata), 25 Polychaeta Families and one Clitellata Family (Naididae) and 30 genera (1 from Clitellata). Based on WoRMS, the Family Naididae is now part of the Order Tubificida, so the dataset has nine orders in total; however, to automatically assign the number of MOTUs per order, the initial eight matched orders were used. Between 1290 and 1361 ASAP clusters were generated from the dataset, with the better partition corresponding to 1350 monophyletic clades with low divergence, which, based on the ASAP/species ratio obtained from the BOLD Annelida dataset ‘DS‐MTWA’ (3131 ASAP/2291 species; 1.36 × ratio) is estimated to correspond to 992 species (1350 ASAP/1.36x; Table 4). Members of the Phyllodocida (the largest Polychaeta Order) revealed a total of 407 MOTUs, whilst ‘Unclassified’ data (this is identified to class or phylum level only) comprised the largest bulk of ASVs, with 1013 MOTUs (Table 4). The Order Eunicida was represented by 114 MOTUs, followed by Terebellida (48), Haplotaxida (45) and the infra‐ and Sub‐Classes Sedentaria and Scolecida (8); and less than 5 MOTUs belonging to Echiuroidea (4), ‘i*ncertae sedis*’ (4), Spionida (3), Sabellida (3) and Amphinomida (2). Whilst no species‐level match was obtained based on the RDP classifier, 679 ASVs matched at least to the genus taxonomic level, retrieving 30 different genera belonging to 244 MOTUs (Table 4; list in Table S3). The total number of MOTUs (1651) for each mentioned major group (e.g., Order level) surpasses the total number of MOTUs for the dataset (1350) due to discordant clades (e.g., one MOTU with two or more different taxonomic identifications). Focusing only on the family level, the Syllidae is the most well‐represented Family in the dataset with 222 MOTUs, followed by Dorvilleidae (61), Polynoidae (55), Eunicidae (32), Phyllodocidae (19), Nereididae (16), with the remaining 20 families having less than five MOTUs (full list in Table S3).

**TABLE 4:** Global DS‐ARMS results (RDP classifier) based on total number of obtained ASVs, number of MOTUs based on ASAP clustering method, estimated species and number of MOTUs identified only to the Class or Phylum (‘unclassified’), Order, Family and Genus.

Metabarcoding dataset DS‐ARMS	
RDP	
No. ASVs	5375
No. MOTUs	1350 (ASAP)
Estimated species^a^	992
No. MOTUs ‘unclassified’	1013
No. Orders	8
No. MOTUs identified to Order‐only	626
No. Families	25
No. MOTUs identified to Family‐only	467
No. Genera	30
No. MOTUs identified to Genus‐only	244

^a^ Based on ASAP/Species ratio using DS‐MTWA dataset (see Section 2).

#### 3.2.1 Taxonomic Ambiguities

A major clade represented by 826 ASVs (15% of the dataset) was highlighted as ‘High taxonomic ambiguity’ (Figure S1), dominated by Phyllodocida (mostly Syllidae), Eunicida (mostly Dorvilleidae) and unclassified data at the Class and Phylum level (Clitellata, Polychaeta and Annelida only) (Figure S1). This clade is composed of MOTUs with < 5 ASVs (usually two) with misidentifications or multiple paraphyletic singleton MOTUs (1 ASV only) amongst those groups. When compared to the MZGdb reference sequences, this clade clustered completely outside of them (data not shown), apart from one sequence—FJ602544 (GenBank accession number), corresponding to *Typhloscolex muelleri* Busch, 1851. Approximately 71% (952) of the total MOTUs are singletons, whilst 4% (50) are classified as discordant. These discordant MOTUs account for 978 ASVs, representing 18% of the entire dataset (Table 5). Discordant MOTUs exhibited varying degrees of taxonomic mismatch across different families (highlighted in red in Figure S1); some were assigned to up to four different families, and in certain cases, even to different classes. Notably, 25 of the 50 discordant MOTUs were located within the ‘High taxonomic ambiguity’ region. ‘Unclassified’ ASVs (identified to the class or phylum level) corresponded to 55% of the entire dataset. The proportion of ASVs identified only to the order, family and genus levels was 12%, 20% and 13%, respectively.

**TABLE 5:** Global species‐level ID results for DS‐ARMS dataset, based on MEGAN, standard single‐nucleotide differences (RDP) and the MetaZooGene Atlas and Database (MZGdb). Quality assessment (singleton, concordant and discordant) of obtained ASAP clusters for DS‐ARMS also displayed. Species lists found in MEGAN and MZGdb are detailed in Table 6.

Metabarcoding DS‐ARMS				
Database species ID consensus		No. MOTUs (ASAP) and quality assessment (RDP)		
		Singleton (No. ASVs)	Concordant (No. ASVs)	Discordant (No. ASVs)
**Database**	**Species‐ level match**	952 (952)	347 (3427)	51 (996)
RDP	0			
MZGdb—Indian Ocean	7^a^ (concordant clusters)			
MEGAN	12 (11 concordant clusters)			

^a^ MOTU match. However, with some intraspecific variation (less 3%–4% K2P).

#### 3.2.2 Species‐Level Matches

**FIGURE 5:**
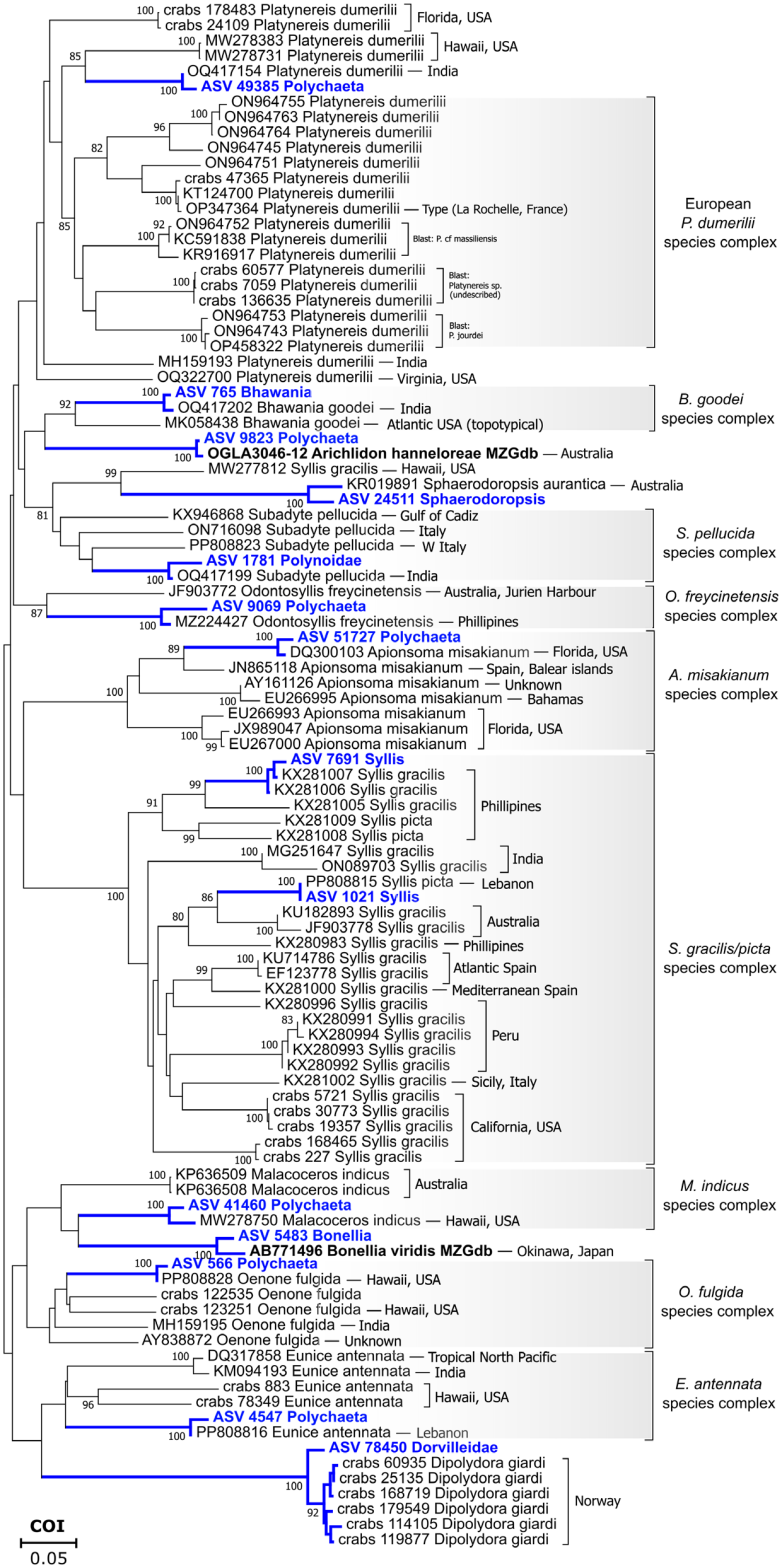
Neighbour‐Joining phylogenetic tree using Kimura 2‐parameter model and based on representative ASVs (DS‐ARMS, highlighted in blue) with available species‐Level identifications (Table 6) and respective sampling locality. MZGdb‐only species highlighted in bold. For the most abundant molecular lineages in the databases, only a maximum of three sequences per lineage was chosen. Only bootstrap (500 replications) values over 85% support are shown. Locality for sequences mined from the CRABS database is unknown, unless stated otherwise.

Although no species‐level matches were obtained using the RDP classifier, a total of 14 unique species‐level IDs were assigned using MEGAN (12 species‐level IDs) and MZGdb (7 species‐level IDs) (Tables 5 and 6). From the latter, all IDs were concordant with the IDs retrieved from the RDP classifier, whilst MEGAN resulted in 11 concordant and 1 discordant cluster (Table 5). From MZGdb, 2 species‐level IDs were not found using MEGAN (Table 6). Amongst the 14 species‐level IDs, four classified as cryptogenic due to the discrepancies between their type locality and the biogeographic origin of the corresponding ASV and database blast; 10 belonged to different species complexes (Figure 5), of which two lineages were native to the Red Sea (Table 6). Some of the species complexes have undescribed molecular lineages with broad geographical ranges (cosmopolitan) from the Atlantic to the Red Sea (e. g., *Apionsoma misakianum* (Ikeda, 1904)) or from the Arabian Gulf to the middle of the Pacific Ocean in Hawaii, USA (e.g., *Malacoceros indicus* (Fauvel, 1928)) (Table 6, Figure 5). A few identifications from MZGdb were linked to sequences outside the Indian Ocean region, for example, *Bonellia viridis* Rolando, 1822 (GenBank: AB771496) from Okinawa, Japan.

**TABLE 6:** Representative ASVs within ASAP clusters (DS‐ARMS; including MOTUs sampled region) and their respective taxonomic match based on MEGAN (only at species level), on MZGdb (Indian Ocean) and ASV RDP match. The latter provides information related to the ASAP discordance report and the respective number of ASVs within each analyzed MOTU (*n*). Type locality for the obtained species‐level IDs and most likely ecological species status in the Arabian Peninsula are also displayed.

Rep. ASVs (Region)	MEGAN	MZGdb – ‘Indian Ocean’	ASV RDP (ASAP cluster discordance)	Species ecological status	Type locality
ASV_5483 (North, Central, South Red Sea)	NA	*Bonellia viridis* Rolando, 1822 GB: AB771496 (Okinawa, Japan)	*Bonellia* (concordant; *n* = 10)	Cryptogenic	Sardinia, Italy
ASV_9823 (North, Central, South Red Sea; Gulf of Oman; Arabian Gulf)	NA	*Arichlidon hanneloreae* Watson Russell, 1998 BOLD: OGLA3046‐12 (Australia, Lizard Island)	Polychaeta (concordant; *n* = 26)	Cryptogenic	Halifax Bay, Australia
ASV_4547 (North, Central Red Sea)	*Eunice antennata*, accepted as *Leodice antennata* Savigny in Lamarck, 1818 GB: PP808816 (Lebanon, Tyre)	NA	Polychaeta (concordant; *n* = 3)	Species Complex, native lineage (Lessepsian lineage)	Gulf of Suez
ASV_566 (North, Central, South Red Sea)	*Oenone fulgida* (Lamarck, 1818) GB: PP808828 (Hawaii, USA)	*Oenone fulgida* (Lamarck, 1818) BOLD: KANBI135‐19 (Hawaii, USA)	Polychaeta (concordant; *n* = 80)	Species complex, native lineage (Indo‐Pacific cosmopolitan lineage)	Red Sea
ASV_1956 (Central, South Red Sea; Gulf of Oman; Arabian Gulf)	*Bhawania goodei* Webster, 1884 GB: OQ417202 (India)	*Bhawania goodei* Webster, 1884 GB: OQ417202 (India)	*Bhawania* (concordant; *n* = 11)	Species complex (Indian Ocean lineage)	Bermuda
ASV_49385 (Central Red Sea)	*Platynereis dumerilii* (Audouin & Milne Edwards, 1833) GB: OQ417154 (India)	NA	Polychaeta (singleton)	Species Complex (Indian Ocean lineage)	La Rochelle, France
ASV_1781 (North, Central Red Sea)	*Subadyte pellucida* (Ehlers, 1864) GB: OQ417199 (India)	*Subadyte pellucida* (Ehlers, 1864) GB: OQ417199 (India)	Polynoidae (concordant; *n* = 25)	Species Complex (Indian Ocean lineage)	Atlantic Europe
ASV_24511 (North, Central Red Sea)	*Sphaerodoropsis aurantica* Capa & Rouse, 2015 GB: KR019891 (Australia)	NA	Sphaerodoropsis (concordant; *n* = 2)	Cryptogenic	Great Barrier Reef, Australia
ASV_9069 (Central Red Sea)	*Odontosyllis freycinetensis* Augener, 1913 GB: MZ224427 (Philipines)	*Odontosyllis freycinetensis* Augener, 1913 GB: MZ224426 (Philipines)	Polychaeta (singleton)	Species Complex (Indo‐Pacific cosmopolitan lineage)	Western Australia
ASV_7691 (North, Central, South Red Sea; Gulf of Oman)	*Syllis gracilis* Grube, 1840 GB: KX281007 (Philippines)	NA	*Syllis* (concordant; *n* = 5)	Species Complex (Indo‐ Pacific cosmopolitan lineage)	Gulf of Naples
ASV_1021 (North, Central, South Red Sea; Gulf of Oman; Arabian Gulf)	*Syllis picta*, accepted as *Syllis violacea* Grube, 1869 GB: PP808815 (Lebanon, Tyre)	NA	*Syllis* (concordant; *n* = 17)	Species complex (Lessepsian‐Arabian lineage)	Port Jackson, Australia
ASV_78450 (Gulf of Oman)	*Dipolydora giardi* (Mesnil, 1893) (Spionidae) CRABS: 25135 (Norway)	NA	Dorvilleidae (singleton)	Cryptogenic	English Channel, France
ASV_41460 (Arabian Gulf)	*Malacoceros indicus* (Fauvel, 1928) GB: MW278750 (Hawaii, USA)	*Malacoceros indicus* (Fauvel, 1928) BOLD: KANBI1434‐19 (India)	Polychaeta (singleton)	Species complex (Indo‐Pacific cosmopolitan lineage)	Gulf of Mannar, India
ASV_51727 (North, Central Red Sea)	*Apionsoma misakianum* (Ikeda, 1904) GB: DQ300103 (Florida, USA)	NA	Phyllodocida (concordant; *n* = 2)	Species complex (Indo‐Atlantic cosmopolitan lineage).	Misaki, Japan

The largest MOTU within DS‐ARMS, composed of 412 ASVs identified as ‘Dorvilleidae’ (Figure S1, highlighted in green and with live photo) based on publicly available data seems to belong to the species *Dorvillea* cf. *similis* (Crossland, 1924) (type locality contained in East Africa) based on our custom Red Sea reference library still in development (private BIN as of 09 April 2025: ‘BOLD:AGQ0405’; https://boldsystems.org/). Additional MOTUs matched species‐level IDs from our private reference library, but either corresponded to undescribed species complexes or were apparently new species. These results and species descriptions will be presented in future publications focused on integrative taxonomy as part of the ongoing effort to improve regional reference libraries in Saudi Arabia.

## 4 Discussion

With the increasing application of DNA metabarcoding techniques to assess biodiversity in tropical coral reefs, a comprehensive understanding of the current status of DNA reference libraries and existing gaps is critical for accurate interpretation of results and informed management decisions. Misinterpretation of molecular‐based assessments can lead to inaccurate biodiversity estimates, potentially resulting in ineffective conservation strategies and inefficient allocation of resources (Weigand et al. 2019; Keck et al. 2022). Over the past decades, a general consensus has emerged that species represent independently evolving lineages of populations or metapopulations (Wiley 1978), though debate persists regarding the point along the divergence continuum at which these lineages should be recognised as distinct species (Hey 2006; Mallet 2008). This allows taxonomy to be integrated with new data and methodologies of population biology, phylogenetics and other evolutionary disciplines (Sites and Marshall 2004; Tan et al. 2010). However, controversy remains regarding the clustering algorithms used in public DNA repositories to define species. Examples of this can be found with the proposed minimalist revision approach based on DNA barcodes and BINs in the description and naming of insect species (e.g., Meierotto et al. 2019; Sharkey et al. 2021) and respective criticism (e.g., Zamani et al. 2021; Meier et al. 2022). Using marine invertebrates as examples, MOTUs clearly overestimate the number of species (e.g., Teixeira, Vieira, et al. 2022; Vieira et al. 2019), with BINs sometimes separating sequences with < 3% mtCOI‐5P divergence into two or more different clusters (e.g., Teixeira, Bakken, et al. 2022). Indeed, evidence of possible undescribed species complexes can also be inferred, in part, by the excessive number of MOTUs, since cryptic species are often detected using molecular data, including annelids (Teixeira et al. 2020; Teixeira, Vieira, et al. 2022; Teixeira et al. 2023).

In this study, we found that the number of BINs in the compiled Annelida BOLD dataset was approximately 1.8 times higher than the number of species, whilst the ASAP clustering method yielded a lower ratio of approximately 1.4. Based on this calibration exercise, and considering that based on the ASAP method our metabarcoding ARMS dataset revealed a total of 1350 MOTUs, we estimate the presence of about 992 distinct annelid species associated with ARMS deployments in coral reefs around the Arabian Peninsula, most of them in the Red Sea. However, this estimate is based on the MOTU/species ratios observed in the DS‐MTWA dataset (see Section 2), which revealed 43% of taxonomic ambiguity (BAGS, Grades C + E; Table 2). Whilst the projected number of species (992) is more conservative than the total number of MOTUs (1350), it remains a rough estimate of the actual number of formal species present in the dataset. Nevertheless, given the available data and methodological frameworks, our metabarcoding dataset detected over one‐third of the Annelida species found in the entire BOLD database (with the applied filters described in the Methods). Moreover, the estimated species richness is approximately twice that reported for the Arabian Peninsula in the OBIS database and 1.3 times higher (Polychaeta only) than the regional checklist published by Wehe and Fiege (2002). Considering that our annelid sequences were obtained exclusively from shallow water coral reefs, not including coastal intertidal, holopelagic, deep sea waters or even sediments (vegetated and non‐vegetated), we cannot extrapolate the total number of annelid species across marine habitats of the Arabian region. However, our results strongly support the idea that reported annelid biodiversity in the region is severely underestimated. This study also reinforces the status of the Arabian Peninsula, in particular the Red Sea, as a hotspot of biodiversity, still largely undocumented (e.g., Sempere‐Valverde et al. 2025) and highlights the urgent need for comprehensive surveys and formal species descriptions through integrative taxonomic approaches (e.g., Elgetany et al. 2022; Teixeira et al. 2024).

### 4.1 Barcode Progress Report in Regional Checklists

Regarding the current data available within the BOLD database, < 24% of the species in the Annelida checklist for the Arabian region and only 30% for the Mediterranean have corresponding DNA barcodes. Similar values were reported by Angulo‐Preckler et al. (2024) for the Red Sea annelids and metazoans in general. These low levels of barcode coverage in the BOLD database are likely a consequence of limited sampling efforts, particularly across large geographical scales, for marine annelids (Weigand et al. 2019). In general, research on marine invertebrates (Duarte et al. 2020) has been considerably lower than on charismatic taxa, such as fish, which have high rates of completion for their inventories (Costa et al. 2012; Oliveira et al. 2016; Cariani et al. 2017). Additional factors likely contributing to the low completion rate of annelid inventories include misidentifications, outdated taxonomy and the prevalence of synonyms. Within the BOLD dataset, 5.9% discordant BINs, 43.1% singletons and 37.2% are classified as Grade D species. Singletons and Grade D species are associated with high uncertainty and low confidence due to the lack of comparable sequences and data from multiple studies. Additionally, a considerable proportion of Annelida species (25.7%) exhibit taxonomic ambiguities (Grade E) and require proper curation.

Despite being an excellent tool for aggregating species records, platforms like OBIS always carry a certain degree of uncertainty. However, they offer a quick way to obtain an approximate list of species and associate diversity across several regions worldwide. Notably, whilst some DNA barcodes exist for species recorded around the Arabian Peninsula, almost none of the sequences in BOLD were collected from this region (only 16 sequences from three species and none from the Red Sea). This underscores the incipient state of the Red Sea barcode library and highlights that further investment is needed to expand sampling across diverse marine habitats, despite the associated logistic challenges. Of the three species barcoded from the Arabian Peninsula ( *Hydroides elegans* , *Hydroides operculata* and *Archinome jasoni*), none were detected in our metabarcoding dataset. The absence of *Hydroides* species is likely due to the amplification challenges commonly faced with the Family Serpulidae, which often requires specific COI primers (e.g., Sun et al. 2012). Despite regional checklists reporting over 50 serpulid species for the Arabian Peninsula (e.g., Wehe and Fiege 2002), no ASVs in our dataset matched this family. *Archinome jasoni*, on the other hand, is a deep sea species, first described in 2012 from hydrothermal vents (Borda et al. 2013), a very distinct habitat from the shallow coral reef environments, making its absence from our dataset expected.

### 4.2 Gaps and Taxonomic Ambiguities in Reference DNA Library Resulted in Biased Metabarcoding‐Based Biodiversity Interpretations

As previously mentioned, DNA barcode reference libraries for marine invertebrates lack a comprehensive biodiversity representation for a given region. Here, the metabarcoding dataset resulted in 1350 MOTUs from 5375 ASVs assigned to Annelida. Yet, a considerable portion of the dataset was classified only at the family and order level, revealing an existing gap for the identification of metabarcodes to the desirable lower taxonomic levels (i.e., genus and species). Moreover, similar to the analysed BOLD dataset (DS‐MTWA), where 43% of the species‐level BINs revealed taxonomic ambiguities (BAGS; Grades C + E), the metabarcoding dataset highlighted the prevalence of sequences belonging to the same MOTU classified to different taxa, often at the family level and occasionally even in different classes. For example, ASVs_92917, ASVs_89953 and ASVs_36258, all correspond to the same MOTU, were assigned to Syllidae (Polychaeta, Phyllodocida), Dorvilleidae (Polychaeta, Eunicida) and Haplotaxida (Clitellata), respectively. We also retrieved a Clitellata genus, usually reported for freshwater habitats (*Chaetogaster*; Table S3) according to WoRMS (https://www.biodiversitylibrary.org/page/37002791) and highly unlikely to be present amongst coral reefs. Furthermore, manual BLASTs in GenBank showed concerning results, with some ASVs showing low identity matches (75%–85%) to Mollusca, despite high sequence query coverage (> 95%). For example, ASV_60396 had an 82.89% match with the tropical snail *Pterocyclos diluvium* Sutcharit & Panha, 2014. In other cases, the sequences are so distinct from those of the GenBank database (e.g., ASV_53785) that the closest match (83.57%) was the cricket *Saga natoliae* Serville, 1838, with only 27% sequence query coverage. These discrepancies could be due to contamination, misidentifications, mislabelling or COI sequence uniqueness. Moreover, when sequences are absent from a particular reference database, the hypervariable nature of COI can hinder its ability to resolve deeper phylogenetic relationships, potentially leading to misinterpretations or ambiguous taxonomic placements.

Pseudogenes may also significantly contribute to the inconsistencies observed in current sequence databases. In our metabarcoding dataset, we detected a considerable molecular diversity, including large paraphyletic clades within the same Order (e.g., Phyllodocida and Eunicida), often associated with high number of non‐singleton MOTUs. Such lack of monophyly has been previously reported for polychaetes (e.g., Zanol et al. 2010; Struck et al. 2011). However, approximately 15% of our ASVs consist of a high number of paraphyletic singleton MOTUs, predominantly between Orders Phyllodocida, Eunicida and unclassified data (up to the class and phylum levels; Figure S1, ‘high taxonomic ambiguity’ section). These inconsistencies may be explained by the amplification of potential pseudogenes, despite the filtering measures applied in our study. This section includes the majority of the MOTUs found for the Family Dorvilleidae (52 out of 61 MOTUs), even though only eight species from this family have been previously reported in the region (Wehe and Fiege 2002). Schultz and Hebert (2022) highlighted the impact of nuclear mitochondrial pseudogenes (NUMTs) in marine metabarcoding, showing that using the standard COI amplicon length (313 bp) could inflate MOTU counts by 21% and increase apparent intraspecific variation by 15%. Authors also stressed that pseudogene detection varies with amplicon length, genomic region and methodology (e.g., eDNA vs. bulk samples) and emphasised the need for improved bioinformatic tools to minimise NUMT‐related artifacts in biodiversity assessments.

### 4.3 Cryptic Complexes May Underestimate the Number of Species in Certain Regions

The presence of cryptic species complexes within Annelida is well documented (e.g., Nygren 2014; Nygren et al. 2018; Grosse et al. 2021; Martinsson and Erséus 2021). In the present study, 17% of the species (15.4% of polychaetes) were classified as Grade C, indicating that each species was represented by multiple non‐paraphyletic BINS (Table 2). This pattern may indicate the presence of cryptic species, suggesting that the actual polychaete diversity in the region (as listed in CL‐MTAP and CL‐MTAWF checklists) may be severely underestimated. A clear example from our DS‐ARMS dataset is a large MOTU assigned to *Dorvillea* cf. *similis*, based on our in‐progress custom Red Sea library. This MOTU does not match any publicly available sequences under the same species name from the Mediterranean (Langeneck et al. 2024) and is identified only to the family level in GenBank or BOLD. The lack of molecular data for species included in dedicated Arabian Polychaeta checklists (e.g., Wehe and Fiege 2002) further suggests that true species diversity is underrepresented due to undetected cryptic lineages.

Figure 5 highlights nine of the 14 species‐level assignments exhibiting a classic cryptic phylogenetic pattern, that is, distinct molecular lineages assigned to the same morphotype. Notably, *Platynereis dumerilii* (Audouin & Milne Edwards, 1833) and *Syllis gracilis* Grube, 1840 show the highest lineage diversity. For *S. gracilis*, Álvarez‐Campos et al. (2017) found five to eight genetically distinct, geographically restricted lineages in the Pacific, whilst Langeneck et al. (2020) reported four divergent lineages in the Mediterranean (type locality), including two distinct morphotypes separated by habitat (brackish water environments vs. coralline algae). Similarly, Teixeira, Langeneck, et al. (2022) reported 10 European *Platynereis* lineages within the *P. dumerilii* complex, redescribing three species ( *P. dumerilii* s.s., *P*. cf. *massiliensis* and *P. agilis* comb. nov.) and formally naming two additional species (*P. jourdei* Teixeira, Ravara, Langeneck & Bakken, 2022 and *P. nunezi* Teixeira, Ravara, Langeneck & Bakken, 2022). Despite these advances, three clades in Figure 5 still have sequences identified as ‘ *P. dumerilii* ’ although they correspond to *P. jourdei, Platynereis* sp. (undescribed) and *P*. cf. *massiliensis*. Furthermore, three additional undescribed lineages in western Italy not detected by Teixeira, Langeneck, et al. (2022) are present within the European cluster (Figure 5; GenBank sequences from Turner et al. 2023, Table S2). These findings highlight the likelihood of even greater undiscovered biodiversity, including unique Red Sea lineages and others continuously emerging in our still private reference library.

The phenomenon of cryptic species is proving to be more widespread and frequent than previously thought not only amongst polychaetes but across other marine invertebrate groups (Sá‐Pinto et al. 2008; Brasier et al. 2016; Vieira et al. 2019; Desiderato et al. 2019). Although the number of sequences for marine invertebrates (e.g., Peracarida, Mollusca, Polychaeta) has increased in genetic databases, dedicated studies combining taxonomists and barcoding resources are still needed to formally describe the new lineages. As noted by Martin et al. (2021), the lack of formal taxonomic descriptions of cryptic lineages may be intrinsically linked to exotic or invasive species. In some cases, introduced species exhibit distinct barcodes in both the invaded area and type locality, suggesting that many potential non‐native species may in fact belong to cryptic complexes (e.g., Table 6, Figure 5). This highlights the difficulties of establishing the invasive status of a species and the need for dedicated studies following an integrative taxonomic approach. This raises the question of whether these species are actually non‐native or just overlooked cryptic complexes. Further analysis of certain species complexes can also reveal molecular lineages not restricted to certain close regions and instead revealing wide geographical ranges (e.g., *A. misakianum* or *M. indicus* , Table 6), which need further analysis regarding their non‐indigenous status in either of the two extremes of its distribution.

### 4.4 Current DNA Reference Libraries Status and Automatic Bioinformatic Pipelines Limit Species‐Level ID Classifications From Metabarcoding Data

Current large‐scale automatic ASV taxonomic assignment is an invaluable tool provided by existing software for efficient and fast use of genetic databases. However, it can be a double‐edge sword due to the lack of regular updates, limited filter options and proper curation of reference libraries. This limitation was revealed in some clades of our metabarcoding dataset, where ASVs belonging to the same MOTU were assigned at higher taxonomic levels (order, class or phylum), whilst only a few were classified at the family or genus level. Also, ASVs may match outdated or misidentified data, even if more accurate barcodes from recent published studies are available, as these tools mostly rely on single‐nucleotide differences for identification. As shown in Table 6, without manually cross‐referencing our ASVs against a FASTA file from the MZGdb, the species‐level matches for seven taxa would have remained unnoticeable, highlighting the limitations of current reference libraries. A noteworthy example is *Arichlidon hanneloreae* Watson Russell, 1998, which did not match any of our ASVs, yet the respective sequence (available only in BOLD since 2012) clustered with our sequences at < 2% divergence (Figure 5). Applying broader taxonomic assignment criteria using MEGAN to the DS‐ARMS (Table 6, Figure 5) increased the number of species‐level identifications. However, MEGAN's limited filtering options restrict the optimal use of available reference libraries. For instance, the software's use of the ‘lowest common ancestor’ (LCA) algorithm to resolve conflicting BLASTn matches—commonly adopted in many metabarcoding studies (e.g., Pearman, von Ammon, et al. 2020)—is understandably conservative. Yet, in cases where misidentifications exist within public databases or where only a few ASVs within a MOTU differ from closely related sequences labeled with outdated or incorrect species names, this approach can significantly underestimate taxonomic resolution. As a result, potential genus‐ or species‐level identifications may be masked, particularly in MOTUs where high similarity exists but is not appropriately resolved due to the LCA conservative assignment. Such inconsistencies combined with lack of consideration for species complexes or their automated flagging (e.g., via the BIN clustering tool; Fontes et al. 2021), can further compromise biodiversity assessments, particularly when regional reference libraries remain incomplete or poorly curated. To address some of these limitations and enhance the interpretation of ASV‐based taxonomic assignments, we advocate for the integration of an optimal post‐processing step in bioinformatic pipelines. This step could incorporate MOTU clustering algorithms guided by user‐defined criteria to improve taxonomic resolution and reduce misassignments. Such criteria could include majority‐rule consensus within MOTUs, exclusion of outlier ASVs (taxonomic‐wise) or divergent COI sequences within MOTUs, prioritisation of published versus unpublished sequences and preference for taxonomic assignments supported by region‐specific reference data (e.g., MZGdb), or detection of cryptic MOTUs (e.g., BAGS, Grade C, Table 2). Given the impracticality of manually BLAST each clade in large datasets, incorporating these refinements would enable a more accurate molecular‐based biodiversity analysis particularly in underrepresented biogeographic regions. Without addressing these issues, metabarcoding results risk being skewed, limiting the potential for accurate biodiversity assessments (see Keck et al. 2022).

## 5 Final Remarks

The importance of curating and improving reference libraries and associated metadata is clearly demonstrated in our analyses. The Annelida database still contains considerable gaps, potential misidentifications, numerous species that are poorly represented globally and regionally, along with other errors in barcode generation. The high number of MOTUs and potential new species identified in our collections around the Arabian Peninsula, coupled with the very limited species‐level taxonomic matches in genetic databases, highlights an unprecedented biodiversity awaiting proper exploration. This underscores the need for future research to provide a more accurate understanding of Annelida biodiversity, as well as other major taxonomic groups.

Large‐scale sampling campaigns, integrative taxonomy and the development of robust regional barcode reference libraries (Hebert et al. 2025) are essential for addressing the shortcoming in existing reference databases and facilitating the accurate identification of both known and undescribed species. Without these improvements, most of the molecular data supporting species hypotheses (Fujita et al. 2012) will remain underused, and biodiversity will continue to be largely unnoticed (Fontaneto et al. 2015). As our results demonstrate, metabarcoding studies within specific habitats cannot fully realise their potential without well‐curated reference libraries, particularly in applications such as biomonitoring (Duarte et al. 2023; Leite et al. 2023). Efforts are currently underway to access biodiversity at the species level for coral reef assemblages and NIS species in man‐made environments (i.e., ports and marinas) in Saudi Arabian waters (e.g., Sempere‐Valverde et al. 2025). These initiatives focus on key marine groups such as annelids, ascidians, bryozoans and crustaceans, aiming to resolve species complexes and update regional checklists to support the development of a comprehensive and reliable reference library for the region. Equally important is the need to revise old sequence data available in public repositories, ensuring that taxonomic identifications are aligned with the most recent and peer‐reviewed classifications. Lastly, to improve accuracy and reliability in biodiversity assessments, bioinformatic tools for metabarcoding assignments should incorporate MOTU clustering algorithms based on refined, user‐defined quality criteria as detailed in the above sub‐section of the Discussion. Implementing such improvements is essential to address the limitations highlighted in this study and to support robust, regionally informed biodiversity assessments.

## Author Contributions

**Marcos A. L. Teixeira:** conceptualization (lead), data curation (lead), formal analysis (equal), investigation (lead), methodology (lead), validation (equal), visualization (equal), writing – original draft (lead), writing – review and editing (lead). **Eva Aylagas:** formal analysis (equal), investigation (supporting), methodology (equal), validation (equal), visualization (lead), writing – review and editing (equal). **John K. Pearman:** formal analysis (equal), investigation (supporting), methodology (supporting), supervision (equal), validation (equal), visualization (supporting), writing – review and editing (supporting). **Susana Carvalho:** conceptualization (supporting), funding acquisition (lead), investigation (supporting), methodology (supporting), project administration (lead), supervision (lead), validation (equal), writing – review and editing (equal).

## Ethics Statement

Sampling of marine invertebrates followed the Institutional Biosafety and Bioethics Committee (IBEC; reference 22IBEC073) approved by the Saudi National Committee of Bio‐Ethics (NCBE; IBEC Registration Number with NCBE, Kingdom of Saudi Arabia: HAP‐02‐J‐042). No animal testing was performed during this study. Sampling and Field Studies: All necessary permits for sampling and observational field studies have been obtained by the authors from the competent authorities and are mentioned in the acknowledgements, if applicable. The study is compliant with CBD and Nagoya protocols.

## Conflicts of Interest

The authors declare no conflicts of interest.

## Supporting information


**Figure S1** Neighbour‐Joining phylogenetic Tree using Kimura 2‐parameters model for DS‐ARMS. All 5375 ASVs are individually shown with the respective taxonomic assignment (RDP). Discordant clades (based on ASAP methodology) highlighted in red. The largest MOTU, identified as ‘Dorvilleidae’, is highlighted in green and match COI sequences of *Dorvillea* cf. *similis* from our custom library in development (live photo for reference). A section corresponding to the ‘high taxonomic ambiguity’ suspected of being pseudogenes is also displayed.

**Table S1** Obtained ASVs and respective number of reads per ARMS and taxonomic identification based on RDP method.

**Table S2** Database COI accession numbers and respective references used in Figure 5.

**Table S3** Detailed list of the obtained Genera and Families from DS‐ARMS. All belonging to Polychaeta, unless stated otherwise.

## Data Availability

All the analyzed datasets, including the metabarcoding ASV alignment ‘DS‐ARMS’ (COI), the worldwide Annelida BOLD compilation ‘DS‐MTWA’ (COI), the COI Annelida sequences from the Indian Ocean (MZGdb) and species complexes used in Figure 5 (‘ASV‐complex’) are publicly available at Figshare (https://doi.org/10.6084/m9.figshare.27938121). The Arabian (‘CL‐MTAP’) and Mediterranean Annelida checklists (‘CL‐MTMS’) based on OBIS, as well as the online version of the Arabian Polychaeta checklist from Wehe and Fiege (2002); revised based on WoRMS: ‘CL‐MTAWF’ are publicly available at Figshare (https://doi.org/10.6084/m9.figshare.27938121) and at BOLD Systems (https://boldsystems.org/) for registered users (CL‐MTAP; CL‐MTMS and CL‐MTAWF, respectively). The online Supporting Information (Tables S1–, S3; Figure S1) is also available at Figshare: https://doi.org/10.6084/m9.figshare.27938121.
