# Supplementary material for: Gaps and Data Ambiguities in DNA Reference Libraries: A Limiting Factor for Molecular‐Based Biodiversity Assessments Using Annelids as a Case Study
Source: Ecol Evol. 2025 Jun 19;15(6):e71544. doi: 10.1002/ece3.71544 (PMC12178944; doi:10.1002/ece3.71544)
Supplement: Supplementary file 3 — Table S2 Database COI accession numbers and respective references used in Figure 5. [file ECE3-15-e71544-s002.docx]

**Gaps and data ambiguities in DNA reference libraries: A limiting factor for molecular-based biodiversity assessments using annelids as a case study**

Marcos A. L. Teixeira, Eva Aylagas, John K. Pearman, Susana Carvalho

**Table S2.** Database COI accession numbers and respective references used in Fig. 5

| **Species** | **Database (COI)** | | | **Reference** |
| --- | --- | --- | --- | --- |
|  | **GenBank** | **BOLD** | **CRABS** |  |
| *Platynereis dumerilii* |  | FLBAR456-17; FLBAR447-17 | 178483; 24109 | Florida Fish and Wildlife Conservation Commission, Fish and Wildlife Research Institute |
|  | MW278383; MW278731 |  |  | Paulay et al., unpublished (2017 MarineGEO Kaneohe Bay BioBlitz) |
|  | OQ417154 |  |  | Ridha et al., unpublished |
|  | ON964755; ON964763;  ON964764; ON964745;  ON964751; ON964752;  ON964753; ON964743 |  |  | Turner et al. (2023) (pre-print) |
|  |  |  | 47365 | Unknown |
|  | KT124700 |  |  | Wäge et al. (2017) |
|  | OP347364 |  |  | Teixeira et al. (2022) |
|  | KC591838 |  |  | Calosi et al. (2013) |
|  | KR916917 |  |  | Lobo et al. (2016) |
|  |  |  | 60577; 7059; 136635 | Unknown |
|  | OP458322 |  |  | Rezzag Mhacene, unpublished |
|  | MH159193 |  |  | Bharathidasan et al., unpublished |
|  | OQ322700 |  |  | Aguilar et al., unpublished (bioblitz) |
| *Bhawania goodei* | OQ417202 |  |  | Ridha et al., unpublished |
|  | MK058438 |  |  | Tilic et al. (2019) |
| *Arichlidon hanneloreae* |  | OGLA3046-12 |  | Museum and Art Gallery of the Northern Territory |
| *Sphaerodoropsis aurantica* | KR019891 |  |  | Capa & Rouse (2015) |
| *Subadyte pellucida* | KX946868 |  |  | Ravara et al. (2017) |
|  | ON716098 |  |  | Mugnai et al. (2023) |
|  | PP808823 |  |  | Toso et al. (2024) |
|  | OQ417199 |  |  | Ridha et al., unpublished |
| *Odontosyllis freycinetensis* | JF903772 |  |  | Aguado et al. (2012) |
|  | MZ224427 |  |  | Verdes et al. (2022) |
| *Apionsoma misakianum* | DQ300103 |  |  | Schulze et al. (2007) |
|  | JN865118 |  |  | Kawauchi et al. (2012) |
|  | AY161126 |  |  | Staton (2003) |
|  | EU266993; EU266995; EU267000; JX989047 |  |  | Schulze et al. (2018) |
| *Syllis gracilis* | KX281006; KX281007;  KX281005; KX281009;  KX281008; KX280983;  KX281000; KX280996;  KX280991; KX280994;  KX280993; KX280992;  KX281002 |  |  | Álvarez-Campos et al. (2017) |
|  |  | DISA447-18; DISA289-18; DISA441-18; DISMA030-17; DISA448-18 | 5721; 30773; 19357; 168465; 227 | Natural History Museum of Los Angeles County |
|  | MG251647 |  |  | Bharathidasan et al., unpublished |
|  | ON089703 |  |  | Mani et al., unpublished |
|  | PP808815 |  |  | Toso et al. (2024) |
|  | KU182893 |  |  | Álvarez-Campos et al. (2015) |
|  | EF123778 |  |  | Aguado et al. (2007) |
|  | MW277812 |  |  | Gustav et al., unpublished (2017 MarineGEO Kaneohe Bay bioblitz) |
| *Malacoceros indicus* | KP636508; KP636509 |  |  | Meissner & Gotting (2015) |
|  | MW278750 |  |  | Gustav et al., unpublished (2017 MarineGEO Kaneohe Bay bioblitz) |
| *Bonnelia viridis* | AB771496 |  |  | Goto et al., 2013 |
| *Oenone fulgida* | PP808828 |  |  | Toso et al. (2024) |
|  | MW277956 | GBMND29604-21 | 123251 | Gustav et al., unpublished (2017 MarineGEO Kaneohe Bay bioblitz) |
|  |  |  | 122535 | Unknown |
|  | MH159195 |  |  | Bharathidasan et al., unpublished |
|  | AY838872 |  |  | Struck et al. (2006) |
| *Eunice antennata* | DQ317858 |  |  | Schulze (2006) |
|  | KM094193 |  |  | Sivaraj et al., unpublished |
|  |  | KANBI169-19 | 883 | Gustav et al., unpublished |
|  | MW278379 |  | 78349 | Gustav et al., unpublished (2017 MarineGEO Kaneohe Bay bioblitz) |
|  | PP808816 |  |  | Toso et al. (2024) |
| *Dipolydora giardi* |  |  | 60935; 25135; 168719; 179549; 114105; 119877 | University of Oslo, Natural History Museum |

**References**

Aguado, M. T., Nygren, A., & Siddall, M. E. (2007). Phylogeny of Syllidae (Polychaeta) based on combined molecular analysis of nuclear and mitochondrial genes. Cladistics 23, 552-564. <https://doi.org/10.1111/j.1096-0031.2007.00163.x>

Aguado, M. T., San Martin, G., & Siddall, M. E. (2012). Systematics and evolution of syllids (Annelida, Syllidae). Cladistics 28, 234-250. <https://doi.org/10.1111/j.1096-0031.2011.00377.x>

Álvarez-Campos, P., Giribet, G., & Riesgo, A. (2017). The Syllis gracilis species complex: A molecular approach to a difficult taxonomic problem (Annelida, Syllidae). Molecular Phylogenetics and Evolution, 109, 138-150. <https://doi.org/10.1016/j.ympev.2016.12.036>

Alvarez-Campos, P., Riesgo, A., Hutchings, P., & Martin, G. S. (2015). The genus Syllis Savigny in Lamarck, 1818 (Annelida, Syllidae) from Australia. Molecular analysis and re-description of some poorly-known species. Zootaxa 4052, 297-331. <https://doi.org/10.11646/zootaxa.4052.3.2>

Calosi P, Rastrick SPS, Lombardi C, de Guzman HJ, Davidson L, Jahnke M, Giangrande A, Hardege JD, Schulze A, Spicer JI, Gambi M-C (2013) Adaptation and acclimatization to ocean acidification in marine ectotherms: an in situ transplant experiment with polychaetes at a shallow CO2 vent system. Philosophical Transactions of the Royal Society of London – B. Biological Sciences 368, 20120444. <https://doi.org/10.1098/rstb.2012.0444>

Capa, M., & Rouse, G., W. (2025). Sphaerodoridae (Annelida) from Lizard Island, Great Barrier Reef, Australia, including the description of two new species and reproductive notes. Zootaxa, 4019, 168–183. <https://doi.org/10.11646/zootaxa.4019.1.9>

Goto, R., Okamoto, T., Ishikawa, H., Hamamura, Y., & Kato, M. (2013). Molecular phylogeny of echiuran worms (phylum: annelida) reveals evolutionary pattern of feeding mode and sexual dimorphism. PLoS ONE, 8, E56809. <https://doi.org/10.1371/journal.pone.0056809>

Kawauchi, G. Y., Sharma, P. P., & Giribet, G. (2012). Sipunculan phylogeny based on six genes, with a new classification and the descriptions of two new families. Zoologica Scripta, 41, 186-210. <https://doi.org/10.1111/j.1463-6409.2011.00507.x>

Lobo J, Teixeira MAL, Borges LMS, Ferreira MSG, Hollatz C, Gomes PT, Sousa R, Ravara A, Costa MH, Costa FO (2016) Starting a DNA barcode reference library for shallow water polychaetes from the southern European Atlantic coast. Molecular Ecology Resources 16, 298–313. <http://dx.doi.org/10.1111/1755-0998.12441>

Meissner, K., & Gotting, M. (2015). Spionidae (Annelida: 'Polychaeta': Canalipalpata) from Lizard Island, Great Barrier Reef, Australia: the genera Malacoceros, Scolelepis, Spio, Microspio, and Spiophanes. Zootaxa 4019 , 378-413. <https://doi.org/10.11646/zootaxa.4019.1.15>

Mugnai F, Costantini F, Chenuil A, Leduc M, Gutiérrez Ortega JM, Meglécz E. 2023. Be positive: customized reference databases and new, local barcodes balance false taxonomic assignments in metabarcoding studies. PeerJ 11:e14616. <https://doi.org/10.7717/peerj.14616>

Ravara, A., Ramos, D., Teixeira, M. A. L., Costa, F. O., Cunha, M. R. (2017). Taxonomy, distribution and ecology of the order Phyllodocida (Annelida, Polychaeta) in deep-sea habitats around the Iberian margin. Deep Sea Research Part II: Topical Studies in Oceanography, 137, 207-231. <https://doi.org/10.1016/j.dsr2.2016.08.008>

Schulze, A. (2006). Phylogeny and genetic diversity of palolo worms (Palola, Eunicidae) from the tropical North Pacific and the Caribbean. The Biological Bulletin, 210, 25-37. <https://doi.org/10.2307/4134534>

Schulze, A., Cutler, E. B., & Giribet, G. (2007). Phylogeny of sipunculan worms: A combined analysis of four gene regions and morphology. Molecular Phylogenetic Evolution, 42, 171-192. <https://doi.org/10.1016/j.ympev.2006.06.012>

Schulze, A., Hipes, J., Borda, E., & Rice, M. E. (2018). Who's who in sipunculans - matching larvae and adults using DNA. In: Proceedings of the Second International Symposium on the Biology of the Sipuncula. Boyle, Michael J. & Kawauchi, Gisele Y. (Eds). Smithsonian Institution Scholarly Press. <https://doi.org/10.5479/si.1943-667X.42>

Staton, J. L. (2003). Phylogenetic analysis of the mitochondrial cytochrome c oxidase subunit 1 gene from 13 sipunculan genera: intra- and interphylum relationships. Invertebrate Biology, 122, 252-264

Struck, T. H., Purschke, G., & Halanych, K. M. (2006). Phylogeny of Eunicida (Annelida) and exploring data congruence using a partition addition bootstrap alteration (PABA) approach. Systematic Biology, 55, 1-20. <https://doi.org/10.1080/10635150500354910>

Teixeira, M. A. L., Langeneck, J., Vieira, P. E., Hernandez, J. C., Sampieri, B. R., Kasapidis, P., Mucciolo, S., Bakken, T., Ravara, A., Nygren, A., & Costa, F. O. (2022c). Reappraisal of the hyperdiverse Platynereis dumerilii (Annelida: Nereididae) species complex in the North Atlantic, with the description of two new species. Invertebrate Systematics, 36, 1017–1061. <https://doi.org/10.1071/IS21084>

Tilic, E., Sermelwall, S. & Bartolomaeus, T. Formation and structure of paleae and chaetal arrangement in chrysopetalidae (Annelida). Zoomorphology 138, 209–220 (2019). <https://doi.org/10.1007/s00435-019-00435-7>

Toso, A., Putignano, M., Fumarola, L. M., Bariche, M., Giangrande, A., Musco, L., Piraino, S., & Langeneck,J. (2024). A revised inventory of Annelida in the Lebanese coastal waters with ten new aliens for the Mediterranean Sea. Mediterranean Marine Science, 25, 715-731. <https://doi.org/10.12681/mms.37998>

Turner, L. M., Madeira, D., Ricevuto, E., Gallucci, A. M., Sommer, U. Viant, M. R., Dineshram, R., Gambi, M.-C., & Calosi, P. (2023). Sibling Species with Different Distributions Around a Co2 Vent Show Proteomic Remodelling Upon Transplantation, While Displaying Unique Metabolite and Lipid Signatures Associated with Their Regimes of Origin (SSRN pre-print). <http://dx.doi.org/10.2139/ssrn.458752>

Verdes, A., Álvarez-Campos, P., Nygren, A., San Martín, G., Deheyn, D. D., Gruber, D. F., & Holford, M. (2022). Molecular phylogeny and evolution of bioluminescence in *Odontosyllis* (Annelida, Syllidae). Invertebrate Systematics, 36, 622-630. <https://doi.org/10.1071/IS22007>

Wäge, J., Valvassori, G., Hardege,J., Schulze, A., & Gambi, M.C. (2017). The sibling polychaetes Platynereis dumerilii and Platynereis massiliensis in the Mediterranean Sea: are phylogeographic patterns related to exposure to ocean acidifcation? *Marine Biology, 164,* 199. <https://doi.org/10.1007/s00227-017-3222-x>
