# Supplementary material for: Gaps and Data Ambiguities in DNA Reference Libraries: A Limiting Factor for Molecular‐Based Biodiversity Assessments Using Annelids as a Case Study
Source: Ecol Evol. 2025 Jun 19;15(6):e71544. doi: 10.1002/ece3.71544 (PMC12178944; doi:10.1002/ece3.71544)
Supplement: Supplementary file 4 — Table S3 Detailed list of the obtained Genera and Families from DS‐ARMS. All belonging to Polychaeta, unless stated otherwise. [file ECE3-15-e71544-s004.docx]

**Gaps and data ambiguities in DNA reference libraries: A limiting factor for molecular-based biodiversity assessments using annelids as a case study**

Marcos A. L. Teixeira, Eva Aylagas, John K. Pearman, Susana Carvalho

**Table S3.** Detailed list of the obtained genera and families from DS-ARMS. All belonging to Polychaeta, unless stated otherwise

| Genera | Families |
| --- | --- |
| *Amblyosyllis* | Ampharetidae |
| *Bhawania* | Amphinomidae |
| *Bonellia* | Bonelliidae |
| *Branchiosyllis* | Capitellidae |
| *Capitella* | Chaetopteridae |
| *Chaetogaster* (Clitellata) | Chrysopetalidae |
| *Cirriformia* | Cirratulidae |
| *Ctenodrilus* | Ctenodrilidae, accepted as subfamily Ctenodrilinae (Cirratulidae) |
| *Eunice* | Dorvilleidae |
| *Eunoe* | Echiuridae |
| *Hesione* | Eunicidae |
| *Iphione* | Hesionidae |
| *Lumbrineris* | Iphionidae |
| *Notopygos* | Lumbrineridae |
| *Paleanotus* | Maldanidae |
| *Palola* | Naididae (Clitellata) |
| *Paradoneis* | Nereididae |
| *Pholoe* | Paraonidae |
| *Phyllochaetopterus* | Pholoidae |
| *Phyllodoce* | Phyllodocidae |
| *Proceraea* | Polynoidae |
| *Protodrilus* | Protodrilidae |
| *Sabellastarte* | Sabellidae |
| *Sabellides* | Sphaerodoridae |
| *Sphaerodoropsis* | Spionidae |
| *Spiochaetopterus* | Syllidae |
| *Syllis* | Terebellidae |
| *Thalassema* |  |
| *Treptopale* |  |
| *Trypanosyllis* |  |
|  |  |
